# Large-scale spatial patterns of small-mammal communities in the Mediterranean region revealed by Barn owl diet

**DOI:** 10.1038/s41598-021-84683-y

**Published:** 2021-03-02

**Authors:** Jan Riegert, Jiří Šindelář, Markéta Zárybnická, Ivan Horáček

**Affiliations:** 1 Department of Zoology, Faculty of Science, University of South Bohemia, Branišovská 1760, 370 05 České Budějovice, Czech Republic; 2 Department of Ecology, Faculty of Environmental Sciences, Czech University of Life Sciences Prague, Kamýcká 129, 165 21 Prague, Czech Republic; 3 Department of Zoology, Faculty of Science, Charles University, Vinicna 7, 128 44 Praha 2, Czech Republic

**Keywords:** Ecology, Zoology, Environmental sciences

## Abstract

Due to mainly opportunistic hunting behaviour of Barn owl can be its diet composition used for assessing local structure of small-mammal community. We evaluated the structure of small-mammal communities in the Mediterranean region by analysing Barn owl diet using own pellets and literature data (85 localities comprising 182,343 prey individuals). Contrary to widely accepted macroecological theory, we found a latitudinal increase of small-mammal alpha diversity, a less distinct west–east increase and lower diversity on islands. The mean prey weight decreased with increasing latitude, while on islands it decreased with increasing island area. The mean prey weight on islands was further negatively affected by mean land modification by human and positively affected by its range. The diet diversity on islands was not affected either by island area or its distance from the mainland. Its composition largely conformed to the main pattern pronounced over whole the region: an unexpected homogeneity of small-mammal community structure. Despite high beta diversity and large between-sample variation in species composition, *Crocidura* (+ *Suncus etruscus*) and murids (*Apodemus*, *Mus*, *Rattus*, in marginal regions partly replaced by gerbillids, *Meriones* or *Microtus*) composed more than 90% of owl prey in 92% of samples. Peak abundances of these widespread species are associated with a dynamic mosaic of dense patches of sparse herb vegetation and evergreen sclerophyllous shrublands interspersing areas of human activity, the dominant habitat of the inner Mediterranean and richest food resource for foraging Barn owls. The respective small-mammal species can be looked upon as invasive elements accompanying large scale human colonization of the region since the Neolithic and replacing original island biota. Our study documented that desertification of the Mediterranean played an important role in shaping inverse latitudinal gradient in diversity of small-mammals that contradicts to widely accepted mecroecological theory.

## Introduction

The Mediterranean region represents one of the most important biogeographical areas of the Western Palaearctic. The region is traditionally considered as the main source for Central-European fauna^1^ and a zone of main Pleistocene glacial refugia for diverse elements of recent communities of mid-European biota^2^. The Mediterranean region is an essential hot-spot of the western Palaearctic biodiversity, including its mammalian fauna^3–7^. For instance, of 222 West Palearctic species of small mammals (41 Eulipotyphla, 122 Rodentia, 58 Chiroptera^8^), 92 species (12, 46, 40, respectively) reach their range margins in the Mediterranean (including core species of the mid-European communities: 11, 33, 18). While 98 species (27, 55, 16) are endemic to that region, only 27 western Palearctic species (2, 4, 21) are distributed beyond the the Mediterranean area. Specificities of the Mediterranean-type communities present, together with the polarity between the eremial and boreal conditions, the most pronounced indexing factors of the Palearctic faunal diversity^9^. Conserquently, the Mediterranean region is often considered as a separate biogeographical sub-region^3^ and in some instances a completely separate region^10^ within the Palaeotropics, exhibiting close affinities to both the Ethiopian and Oriental regions. While regional biodiversity of mammals within the Mediterranean has been frequently studied (e.g.^11–17^, large-scale patterns of small-mammal fauna across the whole Mediterranean region are still not completely comprehended^18^.

In current macroecological theory^19^, the latitudinal diversity gradient (LDG) represents one of most obvious pattern of biodiversity distribution. The LDG, already described by Darwin^20^ and Wallace^21^, predicts a general decrease of species richness with increasing latitudes. Such a pattern has been subsequently confirmed in many and diverse taxa, in both plants and animals, across spatial scales and continents (reviewed by^22^). Thus, it is often considered a universal rule underlying spatial organization of global biodiversity^23^. Yet, at the same time, exceptions exist, reported and explained either by intrinsic specificities of the respective taxa^24^ or by extrinsic effects of regional conditions. Among the latter, several mechanisms have been proposed, including: proximity to large water bodies^25^, desertification^26^, distance of the island from mainland and other cues of vegetation cover disintegration and habitat fragmentation^27^, effects of island biogeography^28,29^ and abiotic factors of geographic isolation^30^, or simply divergent stochastic forces^31,32^. Besides the extensive effects of the long-lasting effects of human colonization^33,34^, all these factors may play a considerable role in the Mediterranean region.

With respect to the Mediterranean region, we suggest that (1) the climate of the region is characterized by pronounced seasonality with prolonged warm summers deficient in precipitations, particularly in the southern part of the region which drives pertinent effects of desertification^33,35^. The extent of desertification, a restrictive limit for temperate taxa, decreases towards the north^36^ and may affect the latitudinal diversity gradient in a considerable way. (2) The Mediterranean region is the area of pronounced paleoendemism^14,37^ and a zone of speciation hotspots associated with the glacial refugia^2,38,39^ in Iberia, Italy, the Balkans, Anatolia, the Levant and Morocco^18^. Also, these factors might considerably disbalance the local diversity patterns especially along a longitudinal gradient. (iii) The large number of islands in the Mediterranean region has resulted in intra-island biotic divergences increasing the overall biodiversity of the region. Despite detrimental effects of human colonization^34,40,41^ island biogeography^28,29^ is nonetheless a prevailing pattern in present-day biodiversity^28,41,42^.

Barn owl (*Tyto alba*) is a nocturnal predator inhabiting a variety of habitat types from rocky landscapes to farmland country in the vicinity of human settlements all over the world except for Antarctica and the northern Holarctic regions^43^. It is particularly abundant across the whole Mediterranean region. The Barn owl feeds on small ground vertebrates, particularly small-ground mammals. It is a typical opportunistic predator whose prey selection is not restricted by further feeding specializations^44^. Thus, its prey remains such as bones and other indigestible parts of vertebrate prey can be used to identify prey species in barn owl pellets. These prey remains provide an almost unbiased picture of the actual composition of local communities of small ground mammals occurring in the foraging area of the respective individual (ca 2–10 km^2^). Moreover, individual owls regularly utilize stable roosting places that allow the collection of pellets in large quantities^45,46^. Consequently, analyses of Barn owl pellets are widely used as an essential source of local faunal information^47–49^. In contrast to expensive and time-consuming conventional trapping (snap traps, live traps or pitfall traps)^50,51^, which is biased by the absence of some trap-shy species (such as *Muscardinus avellanarius* or *Suncus etruscus*)^52^ or incomplete time and habitat coverage, the owl pellet analyses provide a reliable assessment of taxonomic structure and abundance of prey communities which is well balanced both in spatial and temporal respects^43,47,48,53–55^. The long-term studies on Barn owl diet performed within the Mediterranean^45,56^ show relatively small seasonal and annual variation, though locally, it may be influenced by temporal fluctuations in prey availability and ad hoc variations in foraging tactics of individual owl^45^. It can be however expected that such a kind of variation may appear in particular sites with roughly equal probability and related biasing influences can be effectively reduced by increasing sample sizes, which was applied in our study. A comprehensive global study on the Barn owl diet^57^ demonstrated no latitudinal trends in diversity of prey, suggesting a common foraging pattern over the whole Barn owl range and confirmed a possibility to exploit the between-region differences in its diet as a source of comparative information on between-region differences in the structure of prey communities.

**Figure 1:**
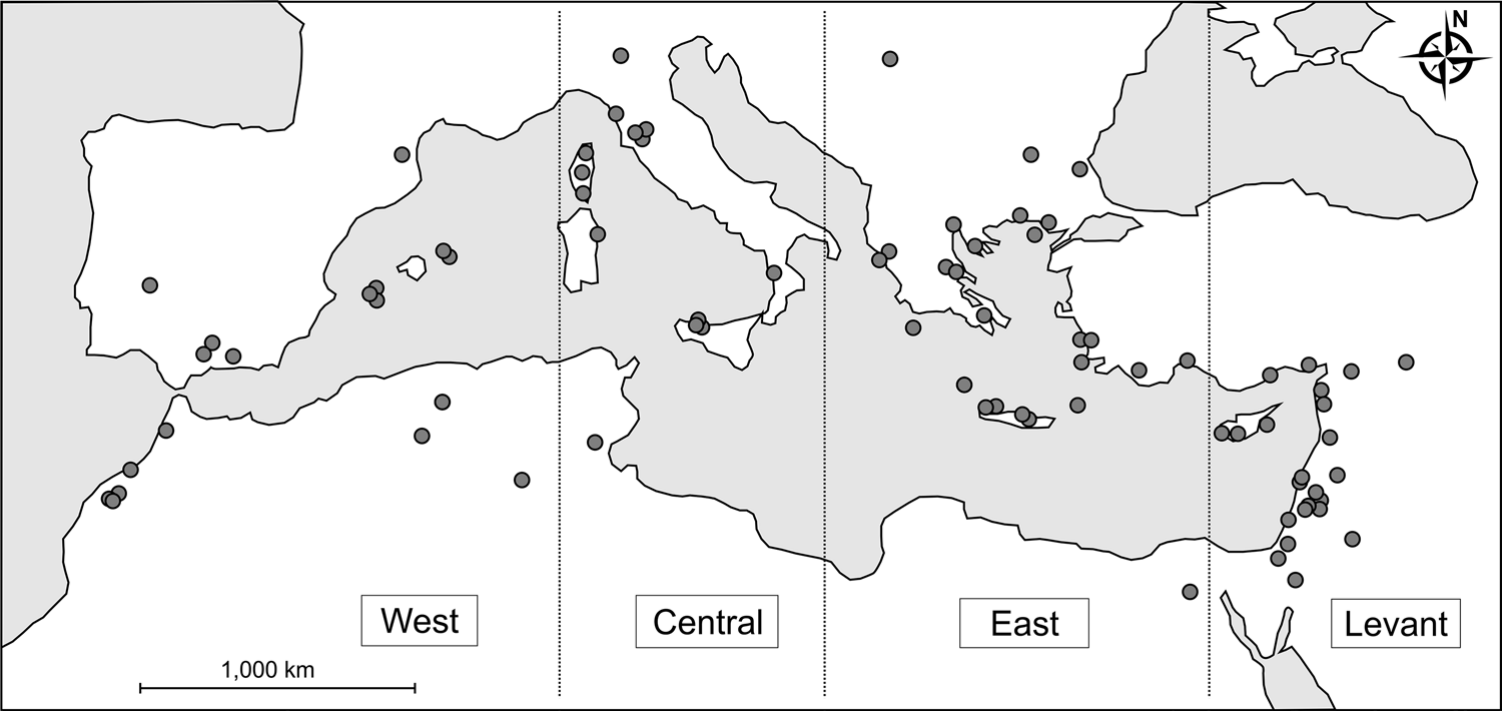
Schematic map of the Mediterranean with localities used for analyses (n = 85) and delimitation of main subregions.

In this study, we examined the diversity patterns of Barn owl diet in the Mediterranean region (n = 85 localities) from the Levant and northern Africa to southern France and Serbia (Fig. 1) both as information on the diversity of the community structure and the distribution of small mammals in that region as well as the diversity of the owl’s foraging strategies. In particular, we tested the following hypotheses: the diversity of small mammals in Barn owl diet within the Mediterranean region would (1) increase with increasing latitude due to the changes in habitat composition along a latitudinal gradient; (2) increase with increasing longitude due to multiple diversity hotspots in the eastern part of the Mediterranean; and (3) be lower on islands compared to the mainland. We further hypothesized that the small-mammal community structure would differ between (4) islands and the mainland, and (5) the main habitats, especially between vegetated and bare habitats. We also tested the effect of land modification by human in surrounding of localities and suggested that (6) land modification would affect the diversity of small-mammal community and mean prey weight. Finally, we hypothesized that the mean prey weight would decrease with (7) increasing latitude and (8) increasing area of the island.

## Results

### The structure of the diet

The total sample of Barn owl diet (85 localities, minimum number of individuals MNI = 182,343) composed of 91.1% mammal (100% of localities) and 8.9% bird (93.0% of localities) individuals. As concerns mammals (see Supplementary Material 1, Table S1 for details), 110 species or subspecies together with further 10 genera of small mammals were determined with dominant contribution by the genera *Mus* (26.7% of total prey items by numbers, 95.4% of localities), followed by *Crocidura* (19.4%, 89.5%), *Microtus* (18.2%, 55.8%), *Apodemus* (12.2%, 64.0%), *Meriones* (2.5%, 33.7%), *Rattus* (2.5%, 77.9%), *Gerbillus* (1.8%, 17.4%) and *Sorex* (1.5%, 18.6%). The number of species composing a sample from locality varied from 3 to 24 (9.64 on average), yet the major bulk of diet consisted of few taxa mentioned above which together composed more than 95% of the diet in 76 (89%) sites and 90% in 80 (94%) sites. Despite considerable between-region and between-sample variation in species composition, the structural characteristics of the diet were largely uniform over the region. *Crocidura* (and *Suncus*) with murids (*Apodemus, Mus* and *Rattus*) formed the eudominant component (63.1% in the total sample, representing more than 80% of prey in 50 (58%) localities). The exceptions represented southern localities, where murids were replaced by gerbillids and *Meriones*, and some mainland localities with increased proportion of *Microtus.*

### Between-region differences in diet diversity

**Figure 2:**
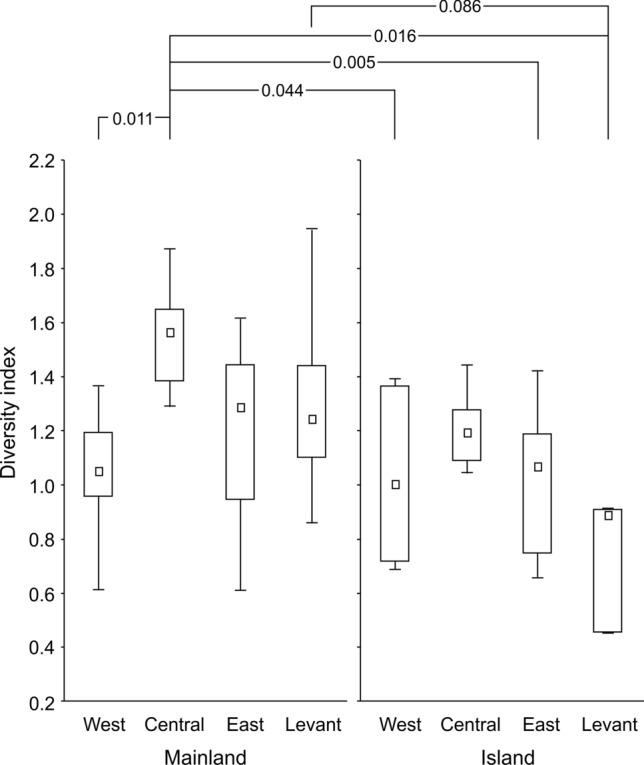
The differences in mammalian prey diet diversity on mainland and island localities regarding to four subregions of the Mediterranean. The numbers above the graph refer to statistical significances and marginal statistical significances (P-values) based on post-hoc tests. Squares—medians, boxes—25–75% of data, whiskers—non-outlier ranges.

We found significant differences in diet diversity among four subregions of the Mediterranean with interaction whether the locality was on the island or mainland (GLMM, explained variability = 50.6%, Chi = 25.2, P < 0.001). In particular, we found the highest diet diversity in the central part of the Mediterranean on the mainland as well as on island localities. Statistical differences were found among mainland locality in the central part of the Mediterranean and three subregions’ island localities from various parts of the Mediterranean. Further, the diet diversity in the Eastern part of the Mediterranean partially decreased that was especially true for the Levant islands. In the Levant part of the Mediterranean, we confirmed marginally significantly lower diet diversity on island localities compared to its subregion mainland localities (Fig. 2). Therefore, we continued with more detailed analyses for subregions that were analogical to analyses on the dataset based on the whole Mediterranean (see “Statistical analyses”). Basic data on diversity patterns in longitudinal subregions of the study area show significantly lower diversity on islands lacking a clear polarization both in longitudinal and latitudinal respects (Supplementary Material 2, Fig. S1, S2). For mainland localities, a peak of diversity seems to appear in the central Mediterranean and towards it, the diversity in both the Western and the Eastern Mediterranean seems to increase. Quite a specific situation appears within the Levant part of the region, which reveals an extremely high variation in diversity patterns and indistinct longitudinal and latitudinal patterning (Supplementary Material 2, Fig. S1). In contrast, visible latitudinal trends appear in the Western, central, and Levant Mediterranean, yet inverse to LDG (Supplementary Material 2, Fig. S2). Detailed analyses showed that the positive relationship between diet diversity and latitude was statistically marginally significant only for the Eastern Mediterranean mainland localities. Similarly, we found significant and marginally significant negative effects of longitude on diet diversity within mainland localities in Eastern and Levant Mediterranean. This is in contrast to the respective patterns revealed in the total dataset (see below). We further found a significant decrease of diet diversity on islands compared to the mainland in the central and Levant part of the Mediterranean. We also found a significant negative relationship between mean prey weight and latitude for mainland localities in the central and Levant Mediterranean. Mean prey weight was also marginally significantly affected by longitude within mainland localities in the Western Mediterranean. Range of land modification had significant negative effect on mean prey weight only within mainland localities in the central Mediterranean. Mean land modification had significant negative effect on mean prey weight only within Eastern Mediterranean (Supplementary Material S3, Table S4–S8).

We further compared between-sample diversity within particular longitudinal subregions and resulting beta diversity values for particular subregions, as well as comparisons concerning a degree of relatedness among the subregions in species composition (Jaccard index) and dominance structure (Renkonen index). The results again reveal distinct differences of the Levant fauna and its closer relations to the fauna of the Eastern Mediterranean. Surprisingly, in its dominance structure it shows certain relations also to the Western subregion’s fauna perhaps due to common sharing of some afro-eremial elements. Note also very high values of beta diversity in all the subregions with a distinct decrease in the central Mediterranean. This is in contrast to the highest mean alpha diversity in the central subregion and strong relations to the Western subregion in species composition (Table 1).

**Table 1:** Indexes of faunal similarity among particular longitudinal subregions in species composition (Jaccard index - upper triangle) and dominance structure (Renkonen index - lower triangle), supplemented with mean values of alpha and beta diversity and values of within-region (gamma) diversity expressed by Shannon diversity index (H').

Subregion	West	Central	East	Levant
West		0.403	0.295	0.182
Central	0.434		0.351	0.241
East	0.206	0.325		0.295
Levant	0.245	0.202	0.404	
Alpha diversity	1.074	1.425	1.163	1.212
Beta diversity	3.475	2.493	3.226	3.289
H'	3.735	3.552	3.652	3.986

### Overall patterns and effect of contextual variables

**Figure 3:**
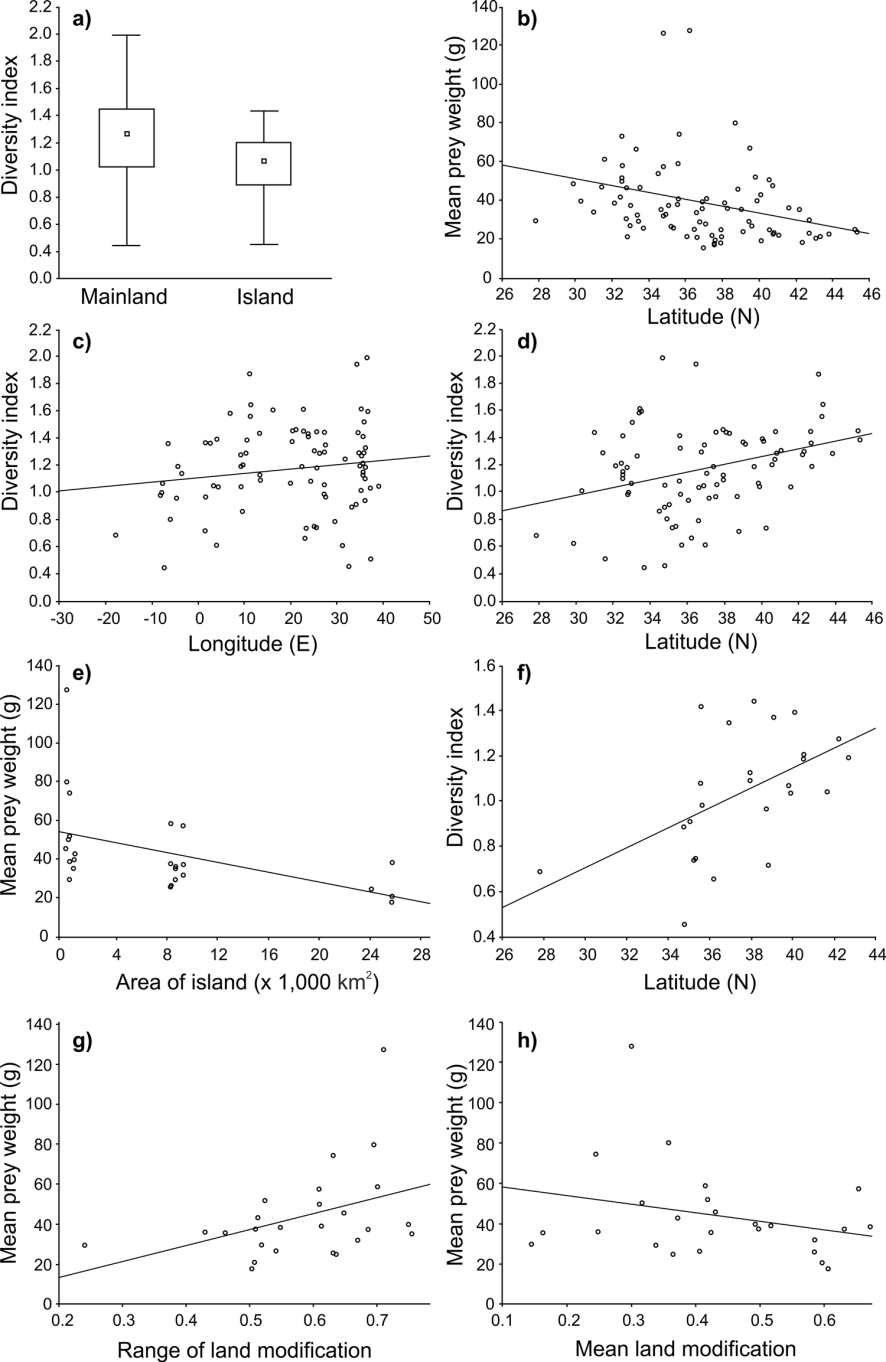
(**a**) Comparison between the diversity of mammalian prey in Barn owl diet on islands and diversity on the continent. The effect of latitude on mean prey weight (**b**) and the effect of (**c**) longitude and (**d**) latitude on mammalian Barn owl prey diversity for the whole dataset (n = 85). (**e**) The effect of the area of the island on mean prey weight and (**f**) the relationship between latitude and mammalian prey diet diversity on islands (n = 25). In boxplot, small squares—medians, boxes—25–75% of data, whiskers—non-outlier ranges.

The diversity of mammalian prey was significantly lower on islands compared to the mainland (Table 2, Fig. 3a), and it was positively correlated with longitude (Fig. 3c) and latitude (Fig. 3d). The relationship between prey diversity and longitude was marginally significant (Table 2). Mean prey weight negatively correlated with latitude (Fig. 3b). When we analysed only data from islands, we found that mean prey weight negatively correlated with the area of the island (Fig. 3e). We also found a negative relationship between the proportion of *Rattus* and the area of island (regression, R^2^ = 0.17, ß = − 0.42, F = 4.86, P = 0.038). Prey diversity on islands was positively correlated with latitude (Fig. 3f). Neither the effect of longitude nor the distance from the mainland was significant (Table 2). We further found a positive effect of range of land modification (Fig. 3g) and marginally significant negative effect of mean land modification (Fig. 3h) on mean prey weight on islands (Table 2). The range of land modification exhibited no significant effect upon dominance of any particular prey item except for a significant negative effect in Chiroptera (regression, R^2^ = 0.37, ß = − 0.61, F = 13.77, P < 0.001), a marginal component of the diet. Also mean land modification did not significantly effect contribution of particular prey items to diet composition composition except for a positive effect upon percentage of the genus *Mus* (regression, R^2^ = 0.18, ß = 0.43, F = 5.13, P = 0.033), which ranks among dominant elements.

**Table 2:** The effect of environmental factors on diet diversity index and mean prey weight for the whole Mediterranean region and for islands only, based on multi-model inference.

Dataset	Dependent variable	Independent variable	Estimate	S.E	z	P
All (n = 85)	Diet diversity	Intercept	0.59	0.60	0.98	0.327
		Island (0/1)	− 0.21	0.07	2.74	**0.006**
		Longitude	0.11	0.13	2.44	**0.079**
		Latitude	0.03	0.01	3.40	**0.001**
		Mean land modification	− 0.03	0.24	0.14	0.890
		Range of land modification	0.06	0.28	0.19	0.848
All (n = 86)	Mean prey weight (g)	Intercept	119.20	28.84	4.07	**< 0.001**
		Island (0/1)	5.42	5.93	0.90	0.368
		Longitude	< 0.01	0.07	0.01	0.992
		Latitude	− 2.28	0.71	3.15	**0.002**
		Mean land modification	− 24.53	16.54	1.46	0.144
		Range of land modification	26.47	20.87	1.25	0.212
Islands (n = 25)	Diet diversity	Intercept	− 0.33	0.82	0.40	0.689
		Longitude	< 0.01	< 0.01	0.10	0.921
		Latitude	0.04	0.02	1.68	**0.094**
		Area of island (km^2^)	< 0.01	< 0.01	0.21	0.832
		Distance from mainland (km)	< 0.01	< 0.01	0.09	0.932
		Mean land modification	< 0.01	0.08	0.04	0.966
		Range of land modification	< 0.01	0.09	0.01	0.991
Islands (n = 25)	Mean prey weight (g)	Intercept	68.22	51.06	1.30	0.194
		Longitude	0.17	0.40	0.40	0.688
		Latitude	− 1.64	1.48	1.08	0.122
		Area of island (km^2^)	< 0.01	< 0.01	2.48	**0.013**
		Distance from mainland (km)	− 0.09	0.06	1.41	0.160
		Mean land modification	− 70.25	36.95	1.83	**0.068**
		Range of land modification	105.50	39.70	2.52	**0.012**

Significant (P < 0.050) or marginally significant (P < 0.100) results are in bold.

**Figure 4:**
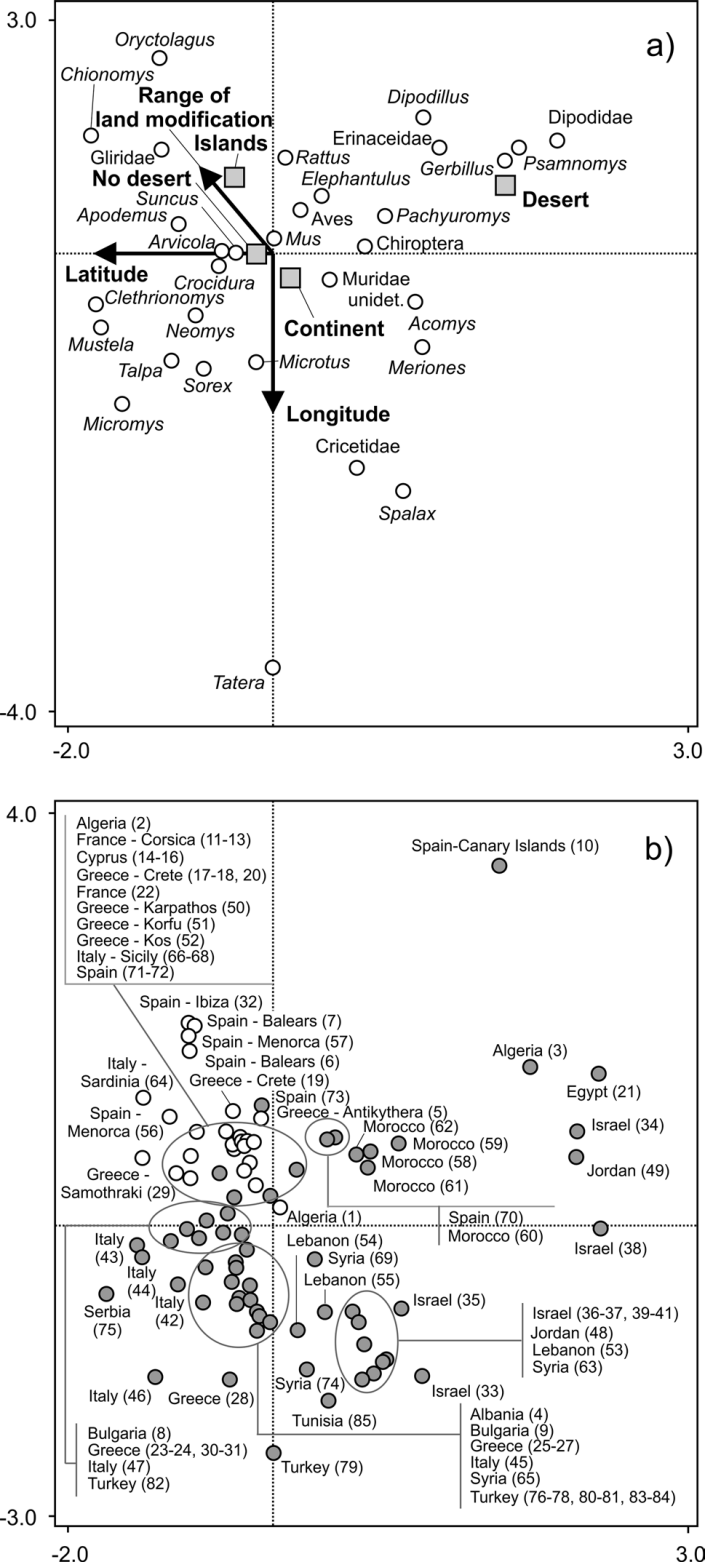
Effect of environmental factors and geographical position on the presence of the main mammalian components (mainly genera) of Barn owl diet in the Mediterranean region (n = 85 localities). The positions of dietary components and factors having a significant effect on their representation (**a**) and sample localities (**b**) within an ordination space are shown. The first and second canonical axes of CCA explain 65.9% of the variability. In graph (**a**), arrows represent geographical trends and range of land modification. Squares represent the island and continental (i.e., mainland) localities and the presence/absence of desert as the main habitat within a locality and circles represent dietary components expressed by log-transformed percentages. In graph (**b**), white circles represent island localities and grey circles represent mainland localities. Numbers in parentheses refer to study numbers according to Supplementary Material 4, Table S10. Note compact clusters of the inner Mediterranean sites and extreme span of variation among sites of the Levant subregion (localities 14–16, 33–41, 48–49, 53–55, 63, 65, 69, 74, 77, 79, 80, 82). The sample 10 (Canary islands) presents the out-group comparison.

Multivariate analysis of total dataset showed that over whole the region dietary composition was affected by latitude, longitude, island/mainland, presence/absence of desert as main habitat and range of land modification (Supplementary Material S3, Table S9). Latitude was negatively correlated with the first ordination axis (correlation coefficient = − 0.68) and longitude was negatively correlated with the second ordination axis (correlation coefficient = − 0.64, Fig. 4a). Range of land modification was negatively correlated with the first ordination axis (correlation coefficient = − 0.24) and positively correlated with the second ordination axis (correlation coefficient = 0.31, Supplementary Material 3, Table S9). Simultaneously, we found increased range of land modification for island localities compared to mainland localities (Fig. 4a). The range of land modification was further slightly positively correlated with latitude (Spearman rank correlation, r_s_ = 0.27, P < 0.050), the correlation with longitude was not significant (Spearman rank correlation, r_s_ = − 0.05, P > 0.050).

**Figure 5:**
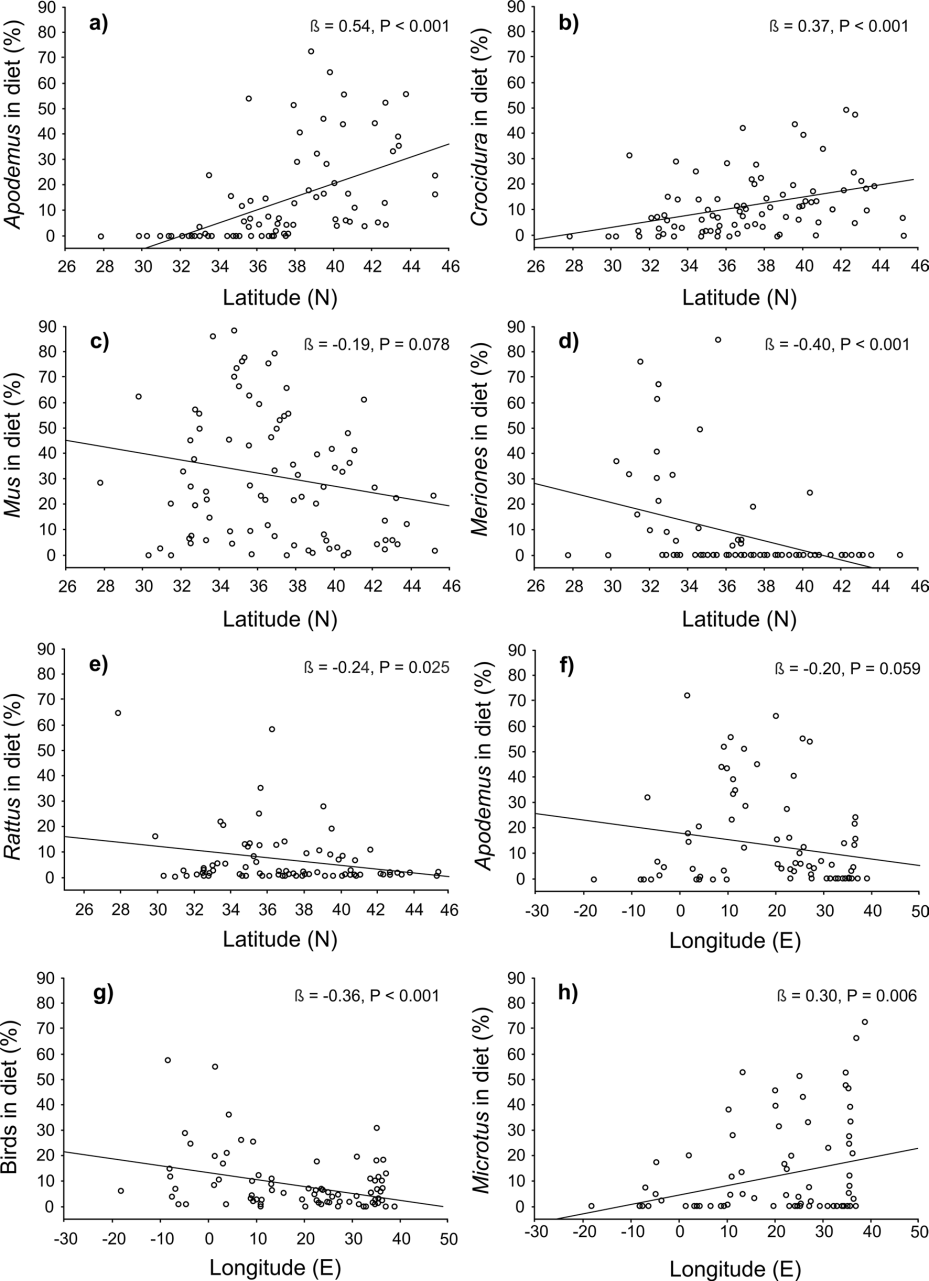
Relationships between latitude and the proportion of *Apodemus* (**a**), *Crocidura* (**b**), *Mus* (**c**), *Meriones* (**d**) and *Rattus* (**e**) in the diet of Barn owl, and relationships between longitude and the proportion of *Apodemus* (**f**), birds (**g**) and *Microtus* (**h**) in the diet (n = 85 localities).

We found significant positive relationships between latitude and the proportion of *Apodemus* (regressions, R^2^ = 0.29, ß = 0.54, F = 34.2, P < 0.001, Fig. 5a) and *Crocidura* in the owl diet (R^2^ = 0.14, ß = 0.37, F = 13.2, P < 0.001, Fig. 5b). Simultaneously, negative (marginally significant and significant) relationships between latitude and the proportion of *Mus* (R^2^ = 0.04, ß = − 0.19, F = 3.2, P = 0.078, Fig. 5c), *Meriones* (R^2^ = 0.16, ß = − 0.40, F = 15.9, P < 0.001, Fig. 5d) and *Rattus* (R^2^ = 0.11, ß = − 0.24, F = 7.9, P = 0.025, Fig. 5e) were revealed.

**Figure 6:**
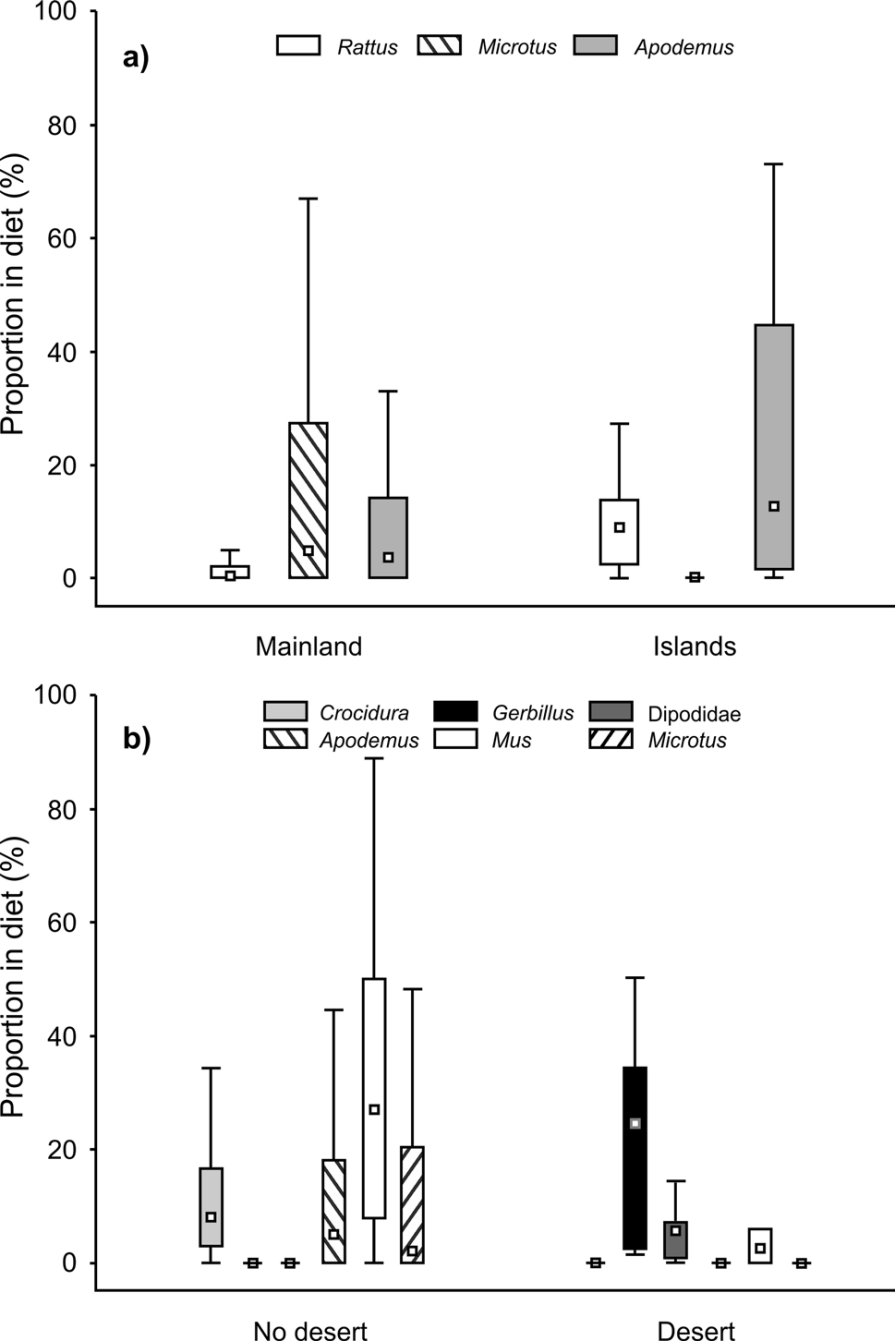
Proportions of selected mammalian prey at (**a**) continental (i.e. mainland) and island localities and (**b**) localities with presence of desert/other habitats. Squares—medians, boxes—25–75% of data, whiskers—non-outlier range.

Significant or marginally significant negative relationships were found between longitude and the proportion of *Apodemus* (regressions, R^2^ = 0.04, ß = − 0.20, F = 3.7, P = 0.059, Fig. 5f) and birds (R^2^ = 0.13, ß = − 0.36, F = 12.6, P < 0.001, Fig. 5g). A positive relationship with longitude was found only with the proportion of *Microtus* voles (R^2^ = 0.09, ß = 0.29, F = 8.0, P = 0.006, Fig. 5h). Western localities were mostly situated on islands (Fig. 4b). Comparisons of dietary composition between mainland and island localities revealed a higher proportion of *Rattus* (Mann–Whitney U tests, U = 286.0, P < 0.001) and *Apodemus* (U = 491.0, P = 0.009) on islands and a higher proportion of *Microtus* on the mainland (U = 347.2, P < 0.001, Fig. 6a). Simultaneously, localities at lower latitudes were often characterized by desert habitat (Fig. 4a). We found a clear separation of taxa occupying this arid environment (Dipodidae, *Gerbillus*, *Psamnomys*, *Elephantulus* and *Pachyuromys*) from other taxa (Fig. 4a). The proportions of *Crocidura* (Mann–Whitney U tests, U = 92.9, P = 0.044), *Gerbillus* (U = 15.1, P < 0.001), Dipodidae (U = 44.5, P < 0.001), *Apodemus* (U = 65.0, P = 0.011), *Mus* (U = 107.0, P = 0.012) and *Microtus* (U = 82.5, P = 0.021) significantly differed between desert and non-desert localities. The proportions of *Gerbillus* and Dipodidae were higher at desert localities compared to other habitats and the opposite was true for the rest of the aforementioned mentioned species (Fig. 6b). Except for degree of desertification (correlated with precipitation environmental variable) and spatial isolation (island/mainland) we found no significant effects of other environmental variables (presence/absence of urban settlement, forest, agricultural land, bush and wetland). Worth mentioning it is particularly in regards to urban and agricultural land variables.

## Discussion

In most regards, our results conform to the patterns revealed by previous biogeographic analyses of European mammals^14,18,58^. They demonstrated: (1) peak diversity in Central Europe contrasting to (2) low alpha diversity in the Mediterranean part of Europe (particularly due to westward decline in representation of widespread Palearctic taxa), (3) extremely high beta diversity and (4) a very high species density (number of species per area unit) in the inner Mediterranean, particularly in the Levant region. Yet the respective analyses were restricted to the area covered by the Atlas of European Mammals^59^, and the topic of species richness in terms of presence of particular species in the Atlas grid system units. We performed similar analyses with completely different dataset extended to the regions not covered by the previous studies. In contrast to the Atlas’ faunal data, our dataset is composed just of a single type source records each representing a single locality, a spatial spot supposedly not exceeding ca 10 km^2^ of owl foraging area. Thus, compared to former analyses, these records can be expected to represent samples of actual local communities of small ground mammals in terms of their real species composition and actual contributions of individual species to community structure. The question is to which degree such expectation is justified or, in other words, to which degree the Barn owl diet can be taken as a reliable source for faunal comparisons. Discussing it, first we should remember the incipient qualities which promote the cosmopolitan distribution of the Barn Owl—its feeding in open grounds and semi-opened habitats and greatly pronounced capability of opportunistic foraging modifying its diet in response to actual availability of local prey^44,45,56^. Correspondingly, in the Mediterranean, the local appearance of Barn owl is clearly confined to the sites providing both suitable nesting possibility (cave entrances and niches in rocky massifs, abandoned human constructions) and foraging ground rich in a mosaic of semi-opened and open habitats hosting abundant communities of small mammals^56^. It seems that owl foraging is restricted to such habitats even in the localities where urban or farmland habitats, forests or wetlands compose the predominant landscape components. This would explain the unexpected absence of these variables’ impact upon diet composition revealed by our study. Also absence of the effect of land modification by human on prey diversity within the Mediterranean suggests that the Barn owl diet is not essentially biased by the proximity of anthropogenic influences. It is essentially composed of the forms composing the communities of "natural" habitats available beyond the sphere of local anthropogenic rearrangements. Despite temporal and local variation reflecting the fine scale habitat differences and other factors influencing prey availability^60^, their overall effects upon the diet composition of Barn owl seem to be of minor importance only on a large scale. For all these reasons, the above expectation concerning reliability of Barn owl diet analyses for faunal comparison seems to be well substantiated. Hence, we strongly believe that the dataset we analyzed provides a robust source both for a comparative study on the owl diet and for quantitative analyses of large scale biogeographic patterns of mammalian community structure.

We found a significant latitudinal scaling of diversity within the Mediterranean region both in owl diet (expressed in terms of phenotype categories) and species alpha diversity of small mammal communities. Yet, it exhibited an inverted pattern of LDG contradicting the common rule of diversity decline with increasing latitude. Of course, the inverse LDG pattern of diversity increase with increasing latitude is perhaps not too exceptional. For example, it was found in small mammals in the realm of the whole Asia^61^ similarly like in other groups^62^ or in the diversity of bird communities on the northern continents, which was explained by seasonality effects providing temporal superabundant summer resources in northern latitudes^63^. Climatic factors also play a significant role in latitudinal resource scaling in the Mediterranean region. The southern areas of the Mediterranean stay under direct influences of the north-African and Arabian deserts, which produce obvious restrictive effects upon prey diversity both in local and regional respects. Nevertheless, even the northern parts of the region, where the contribution of desert elements is negligible, exhibit the pattern suggesting that the lower latitudes offer fewer opportunities for diversified local mammal communities than higher latitudes that is in contradiction to LDG assumptions. In a search for the reasons, at least three factors are to be taken into account: (1) the land cover in lower latitudes is distinctly smaller both due to the peninsular pattern of mainland margins and numerous islands with correspondingly lower mammal diversity^14,42^, (2) the inner Mediterranean and particularly the islands or shore areas have been exposed since late Neolithic to steady anthropogenic impact which caused multiple extinctions of local endemics^34,40^. Simultaneously, large-scale habitat rearrangements promoted the expansion of open ground inhabitants and spread of invasive elements^7,41,64^. Finally, (3) the respective anthropogenic changes with extensive land degradation^65^ might even strengthen the incipient latitudinal scaling of climatic currents responsible for aridisation tendencies throughout the region^35^. Towards the north, the environment dramatically changes due to increasing precipitation and the greater proportion of continental habitats associated with a higher representation of forests and lower representation of arid habitats. It is beyond the scope of this paper to discuss which of these factors might have played a decisive role. However, all of them might contributed in synergy to a decrease in the diversity of local mammalian communities of the southern part of the region and to the inverted latitudinal trend of prey diversity in the Mediterranean region.

A non-trivial outcome of our study is that despite excessive values of beta diversity and large differences in species composition among particular samples and latitudinal areas, the main pattern of the Barn owl diet and core structure of mammalian communities is nearly uniform over most of the region. It is composed of few taxa (*Crocidura* + *Suncus, Apodemus, Mus,* and *Rattus*) that can be in common characterized as the generalists capable of opportunistic response to variation in both feeding and habitat resources. All these taxa exhibit a pronounced capacity for rapid colonization of mosaic environment, densely alternating patches of sparse herb vegetation with evergreen sclerophyllous shrublands—maquis (machia, matoral and garigue), the most characteristic vegetation formation of the Mediterranean region^66^. This habitat mosaic, prone to invasion species, presents a dynamic complement of the Mediterranean vegetation to deforestations, pasture and other antrophogenic influences lasting here from the beginning of the Neolithic revolution^33,65–68^. Its widespread distribution over the Mediterranean region is in perfect accord with surprising invariance in the core of small mammal communities.

In our total sample, the diversity of small mammals showed a significant increase from western to eastern areas, while the proportion of birds in the diet significantly decreased towards eastern areas. The latter partially disagrees with Roulin’s^69^ findings, showing Barn owl consuming more bird prey in Eastern Europe. However, his survey included a large number of samples from Central and even Northern Europe. Owls, including Barn owl, usually prey on birds during scarcity of small-mammal prey^70^, which can occur more frequently in the western than in the eastern part of the region. This is because of a stronger reduction of vegetation cover or lower degree of faunal saturation in the west compared to the Eastern Mediterranean, resulting from different faunal and climatic history^11,12,71,72^. Moreover, western localities in our sample were partly situated on islands with low mammal diversity suggesting that the longitudinal trend in prey diversity might also reflect the distribution of islands within the Mediterranean.

Small-mammal diversity was significantly lower on islands compared to the mainland, but did not vary with the area of the island. Decreased Barn owl diet diversity on islands compared to the mainland has been recently confirmed throughout the Barn owl range^57,73^. Decreased small-mammal diversity due to island isolation was also reported in other studies from the Mediterranean^14,74,75^. Yet, against many studies performed on island biota (e.g., Lesser Antilles^76^, South-eastern Asian islands^77^, tropical Pacific islands^78^, Japanese islands^79^, and Elba and Capraia islands^80^), we found no clear relationships between the species diversity and the area of the island. This fact can be ascribed to extensive extinctions of original endemic biota in particular islands replaced by a cluster of modern invaders common to the whole Mediterranean introduced via human activity^7,34,40,41^.

Besides variation specific to particular islands, we found some common difference in community structure of small-mammals between islands and the mainland. The genus *Microtus* presents a subdominant element in mainland localities and except for Sicily it absents on islands, while the genera *Rattus* and *Apodemus* reach peak of their dominance just on islands. The genus *Rattus* (mainly Black rat *R. rattus*) was the third most important diet item in terms of biomass and occurred at 78% of all localities and 96% of island localities. In the Mediterranean region, Black rat forms abundant feral populations originating from the multiple introductions being a frequent companion of man during his ship journeys during colonization and trade since the early Middle Ages^64,81^. Invasions of Black rat together with Brown rat *Rattus norvegicus* (which still does not form feral populations on the Mediterranean islands) and House mouse *Mus musculus* are often associated with declines or extinctions of a large number of indigenous vertebrate species and with ecosystem changes on islands^82^. These invasive mammals, especially Black rat, overpower native (and often endemic) species as a result of their large somatic parameters and trophic adaptability^83,84^ and their ability to withstand living in high-density populations with low risk of going extinct when living in small populations on small islands^85^. All these characteristics make Black rat one of the most successful invaders and essential agents of diversity decrease of the prey community on islands.

Within the genus *Apodemus*, Wood mouse *A. sylvaticus* (but see Supplementary Material 1, Table S1) was the most abundant species and was found at 38.4% of all localities and 76.0% of island localities. It has been recorded in almost every locality of the Western and central Mediterranean region, while on Cyprus it was substituted by House mouse. The highest proportions of Wood mouse were found on islands (Samothraki 54.8%—this study; Corsica 40.9%^86^; Sardinia 40.4%^87^; Balears 66.3%^88^). In the central and eastern part of the Mediterranean region was present the largest representative of the genus Broad-toothed field mouse *A. mystacinus/epimelas*, forming 48.1% of prey individuals on Karpathos. Yellow-necked mouse *A. flavicollis* was only present at more humid mainland localities in the northern part of the area (19.1% of all localities).

The genus *Microtus* formed an important dietary component in mainland localities (74% of mainland localities vs. 16% of island localities: Sicily, Corfu, Samothraki). Except for Sicily inhabited by a dense population of *M. savii*, the other two island records come from shelf islands close to mainland shore. In Corfu, which least distance to shore is just 2 km, the pellet sample included even a mole, which otherwise, similarly like subfossorial voles not appears on islands^40–42^, except those neighboring the mainland shore^89^. In contrast to the genera *Rattus* and *Apodemus*, no species of the genus *Microtus* exhibit characteristics of an euconstant element. The most frequent European species, *M. arvalis* (including *M. levis* - a vicariant sibling species in the eastern part of the Eastern Mediterranean), appeared within the total sample at only 6.9% of localities including northern continental parts of the Mediterranean region (northern Italy, France, Serbia and Turkey). *M. guentheri* restricted to the eastern part of the Mediterranean region was more widespread (15% of localities), while *M. duodecimcostatus,* the West Mediterranean endemic appeared in mere 5.8% of localities. The occurrence of each of these species in the diet largely coincided with the species’ geographical range^59^.

As predicted, distinct habitats within the region were occupied by different groups of genera. The multivariate correspondence analysis clearly separated the sandy- and open-habitat specialists Dipodidae, *Gerbillus*, *Psamnomys*, *Elephantulus* and *Pachyuromys* from other taxa. It has been experimentally proved by artificial removal of dense shrubs from sand dune areas that these newly emerged bare habitats become soon colonized by representatives of gerbils *Gerbillus* and jirds *Meriones*. On the other hand, species like White-toothed Shrew, House mouse, and Black rat avoid such habitats^90^. Our results showed that *Meriones* jird was not categorized as a strictly desert taxa, since it also occupies semi-arid grasslands and agricultural fields in Turkey^91^, Syria^92^, and Lebanon (this study). In contrast to *Meriones*, both the genera *Gerbillus* and *Jaculus* specialize in occupying open habitats without vegetation^16^. We propose that only these two genera can be classified as strictly desert taxa within the Mediterranean region.

We found that mean prey weight decreased with increasing latitude and negatively correlated with the area of the island. The lower mean prey weight documented at higher latitudes was caused by a decreasing proportion of larger prey items (*Rattus* and *Meriones*) in Barn owl diet within these areas. The complete absence of the genus *Meriones* at localities north of 40.5° N corresponds well with its known geographical range and habitat requirements for drier sandy and clay habitats^16^. As already mentioned, the occurrence of the introduced genus *Rattus* (especially Black rat, *R. rattus*) in the area of the Mediterranean basin (in particular on islands) is rather crucial and raises significantly the mean weight of Barn owl prey. A decrease of mean prey weight with increasing latitude was contributed by increasing proportion of the small-sized members of *Crocidura* (mainly *C. suaveolens*, *C. russula* and *C. leucodon*), the forms demanding a dense herb vegetation associated with increased precipitation in northern latitudes. The negative relationship between the mean prey weight and the area of island reflects a reduced dominance of *Rattus* in large islands compared to smaller forms such as *Apodemus*, *Mus* or *Microtus* in Sicily. Finally, we found that mean prey weight on islands was positively correlated with range of land modification by human and negatively corelated with mean land modification by human. A negative relationship between mean land modification and mean prey weight on islands can be significantly contributed by increased proportion of genus *Mus* on islands with increased mean land modification.

To conclude, we verified instant macroecological predictions on the distribution patterns of small mammals in the Mediterranean region using the method of Barn owl pellet analyses. Species diversity and mean weight of small mammals in Barn owl diet in the Mediterranean follow latitudinal and to a lesser extent also longitudinal gradients. Some general patterns such as the effect of the island on the diversity and weight of small mammals were consistent with established ecological theory. On the other hand, the latitudinal gradient in the diversity of small mammals was in contrast to established theory with certain difference between the longitudinal subregions. We suggest that the patterns we found in small-mammal distribution resulted from synergic effects of latitudinal climatic variation with desertification in the south, geographic specificities (islands, refugial areas on peninsulas etc.), and historical anthropogenic effect influencing excessively the Mediterranean biota continuously throughout the human’s postneolithic history.

## Methods

### Dataset

We used datasets (abundances of particular species, contextual variables) from 85 localities (samples) between 29.8° and 46.1° N and between 18.0° W and 39.0° E, covering an area of over 5,000,000 km^2^ representing the core (especially NW) area of the Mediterranean region in the sense of^10^. Of these samples, 14 were based on our pellet analyses and 71 were based on pellet analyses from literature sources. In total, we recorded 182,343 prey individuals (9,336 inds. based on our pellet analyses and 173,475 inds. based on literature sources), of which 166,063 (91.1%) were mammals and 16,280 (8.9%) were birds. For purposes of the between-region comparisons, the dataset was further subdivided into groups of the West Mediterranean (-18° to 8° E), central Mediterranean (8° to 18° E), East Mediterranean (18° to 32° E), and Levant (32° to 40° E, Fig. 1).

### Pellet analysis

We collected Barn owl pellets at 14 localities in Cyprus, Crete, Lebanon, Karpathos, Corfu, Turkey, Greece and Serbia between 1988 and 2010 (Supplementary Material 4, Table S10). In most instances, the samples were taken just at a single ad hoc visit of the site. Particular sites have been spatially well delimited (mostly a single cave entrance, rocky overhang and similar natural sites, quite exceptionally a space within a single human construction), in most instances they represented a single nest site often used regularly for relatively long period (several years or so). The major bulk of the material composed of intact pellets. Complete pellets were dissolved individually in a 5% NaOH solution and then bones and other prey remains were sorted^49^. The number of prey items from each locality was determined by the presence of unique structures, such as skulls and pairs of mandibles^46^. A total of 8,489 mammals and 847 bird individuals (mean ± s.d., 667.9 ± 749.6 inds. per locality) were recorded in the pellets. Mammalian prey items were identified based on cranial and dental characters with the aid of stereomicroscope and determination manuals^13,16,75,93^. Bird species were not determined. In few sites of our samples where bird remains appeared in larger numbers, they belonged in most instances to house sparrow (*Passer domesticus*). Estimation of the mean weight of individual mammalian species was based on literature sources^13,16,75,93^. The mean prey weight in particular samples was calculated as a sum of the mean weight of each particular species multiplied by their relative contribution. The mean weight of birds was calculated based on average from mean bird weight from studies, where at least 500 bird individuals were identified^86,94–96^.

### Published datasets

Besides analyses of the samples collected in the field by IH, we also conducted a literature survey of Barn owl dietary composition for further 71 localities (samples), comprising 158,040 mammal and 15,435 bird individuals (mean ± s.d.; 2433.3 ± 4491.1 inds. per locality), from studies published between 1947 and 2015. The dataset included pellet analyses of samples from Algeria, Egypt, France, Greece, Morocco, Spain, Israel, Italy, Jordan, Bulgaria, Syria, Turkey, and Tunisia (Supplementary Material 4, Table S10). The sample from Canary islands was included for out-group comparisons. We included only samples that were precisely geographically defined and contained a detailed account of the species composition of small mammals and/or the contribution of higher taxonomic categories (mammals, birds, etc.).

### Environmental factors

Each locality was characterized by geographical coordinates using a WGS 84 system. For each locality we noted whether it was mainland or island, with or without vegetation, and with or without forest cover. We also collected data on precipitation averages for each locality from relevant websites (based on the worldweatheronline.com database). Each locality was described by the presence or absence of the following main habitats: urban settlement (12.9% of localities), forest (35.3%), agricultural land (78.8%), desert (5.9%), bush (7.1%), and wetland (3.5%) within an approximate radius of 1 km around the collection site based on the pellet collector’s site description or personal notes and descriptions in references. Note that one locality has been often described by two main habitats (Supplementary Material 4, Table S10). The area of islands ranged from 2 to 25,711 km^2^ (mean ± s.d.; 7651 ± 8,610 km^2^), and the distance of islands from the mainland ranged from 3 to 319 km (mean ± s.d.; 103.7 ± 77.4 km). Land modification by human within a radius of 10 km around each study mid point was gained from NASA database Socioeconomic data and application center (SEDAC) using a grid 1 × 1 km^97,98^. In particular, we used mean (0–1) and range of land modification within the buffers for further analyses.

### Statistical analyses

For each locality (sample), we calculated the Shannon-Weaver diversity index^99^ based on the percentage contribution of all particular species composing the sample. This became an indexing characteristic of the sample and an essential input variable for further analyses.

Besides that, in order to exclude a possible bias of instable species identity, between-region differences in taxonomic status of local vicariant taxa and sibling species, we analysed the prey diversity also in terms of common phenotype categories, i.e., genera of regularly represented mammalian forms or higher taxa for rarely appearing elements (Chiroptera, Cricetinae, Erinaceidae, Gliridae etc.) and the group not identified at the species level (birds). Despite quite different numerical values both the diversity measures exhibited quite a tight correlation (R^2^ = 0.905). A comparison of community structure among subregions was done using Jaccard and Renkonen index.

The effect of the various environmental factors on mammalian prey diversity and mean prey weight for islands (n = 25) and all localities (n = 85) was tested in R 3.4.4 software^100^ using a multi-model inference approach (model.avg function in MuMIn package^101–103^) based on AIC differences. We used the following factors for building candidate GLMM models (glmer function in lme4 package): island/mainland (0/1), longitude, latitude, mean land modification (0–1) and range of land modification within a buffer. For analyses of island data, we also tested two factors: area of the island (km^2^) and distance from the mainland (km). As mean precipitation was negatively correlated with longitude (Spearman rank correlation, r_s_ = − 0.40, P < 0.050) and positively with latitude (r_s_ = 0.52, P < 0.050), we excluded this factor from analyses because of collinearity. For each of four dependent variables (diversity index for island localities, diversity index for all localities, mean prey weight for island localities and mean prey weight for all localities) we built null and 12–16 alternative models with a Gamma distribution of dependent variables and the number of collection sites within a particular study multiplied with the number of years when the material was gathered as a random factor. For island analyses, we also added the identity of the island as a random factor. The reasons for this were to eliminate possible biases caused by different sample sizes and pseudoreplications that may arise when more than one sample were located at the same island. We created the models with each factor alone, and then we subsequently added other factors. Here, we show results for the full average procedure (i.e., the results from the comparison of all models). The AIC values of the compared models for each type of GLMM are available in Supplementary Material 3, Table S2, S3. Similarly, we performed these analyses also for Mediterranean subregions separately (Supplementary Material 3, Table S4–S8).

Multivariate data on dietary composition (mainly at genus level) were analysed using Canonical correspondence analysis (CCA) in Canoco 5 software^104^. Proportions of taxa in Barn owl diet were log-transformed prior to analysis and the number of collection sites within a particular study multiplied with the number of years when the material was gathered was used as a covariate. We tested the effect of latitude, longitude, island/mainland (0/1), urban/rural habitat (0/1), forest/no forest habitat (0/1), agricultural/no agricultural land (0/1), desert/no desert (0/1), bush/no bush (0/1), wetland/no wetland (0/1), mean land modification (0-1) and range of land modification using a forward selection. Statistical significances were obtained by Monte-Carlo permutation tests (n = 999 permutations).

Analyses on the proportion of particular diet items in relation to island/mainland and desert/no desert were performed using the Mann-Whitney U test. The relationships between proportions of a particular main prey item and latitude or longitude were calculated using regression. These tests were carried out using Statistica 13 software^105^.

## Supplementary Information

Supplementary Information 1.

Supplementary Information 2.

Supplementary Information 3.

Supplementary Information 4.

## Data Availability

All data are available in Supplementary Material 1 and 2, Tables S1 and S10.
