# Supplementary material for: Large-scale spatial patterns of small-mammal communities in the Mediterranean region revealed by Barn owl diet
Source: Sci Rep. 2021 Mar 2;11:4985. doi: 10.1038/s41598-021-84683-y (PMC7970837; doi:10.1038/s41598-021-84683-y)
Supplement: Supplementary file 1 — Supplementary Information 1. [file 41598_2021_84683_MOESM1_ESM.pdf]

Supplementary Material 1

Inferring large-scale spatial patterns of small-mammal communities in the Mediterranean region revealed by Barn owl diet

Jan Riegert, Jiří Šindelář, Markéta Zárybnická & Ivan Horáček

Small mammals in Barn owl diet

The genus *Mus* was largely represented by *M. musculus* (34.2%), *M. spretus* (16.3%) and *M. macedonicus* (10.8%), but with a large proportion also remaining unidentified (38.7%). Within the genus *Crocidura*, the most common species were *C. suaveolens* or *gueldestaedtii* according to Bannikova et al. (2006) (44.9%), *C. russula* (32.6%) and *C. leucodon* (20.7%). Nine species of the genus *Microtus* were recorded, but the most frequent were *M. arvalis* incl. *levis* (23.2%), *M. guentheri* (18.5%) and *M. duodecimcostatus* (7.0%). Among the genus *Apodemus,* seven species were recorded, of which the most dominant was *A. sylvaticus* (43.9%). Yet, regarding extensive overlaps in most morphometric characters among all species of the *Apodemus* subgenus *Sylvaemus* (i.e. *sylvaticus, flavicollis, whitherbyi, uralensis*) - comp. e.g. Kryštufek &Vohralík (2010), Knitlová & Horáček (2017), in more samples of the central and Eastern Mediterranean the proposed species identifications within that group are to be looked upon rather as just a provisional. The genus *Meriones* was represented by seven species, with the most common being *M. tristrami* (38.2%). Among the genus *Rattus*, the most common species was *R. rattus* (74.1%), with *R. norvegicus* being fairly scarce (5.7%) and remaining individuals not determined (20.2%). Ten species of *Gerbillus*, with the most dominant being *G. campestris* (57.6%), were also found. We recorded three species of *Sorex,* the most common being *S. araneus* (57.0%) and *S. minutus* (40.9%). The other mammalian taxa constituted 9.1% of prey items. These taxa included Chiroptera (0.2% by numbers, 32.6% of localities), which was significantly represented only in Longoz, western Turkey (10.8%, five species) and in Harran, southeastern Turkey (5.1%, two species).

Larger prey was represented by Erinaceidae (< 0.1% by numbers, 10.6% of localities) and European rabbit *Oryctolagus cuniculus* (< 0.1%, 1.8%). The smallest prey (ca 2g) was represented by Etruscan shrew *Suncus etruscus* (1.4%, 58.0%). At several localities in Israel, Lebanon and Turkey, individuals of the genus *Spalax* were recorded, these most often being *S. ehrenbergi* (0.1%, 12.9%).

By biomass, the genus *Microtus* dominated the dietary composition (21.5%), followed by *Mus* (17.7%), *Rattus* (16.1%), *Apodemus* (11.9%) and *Meriones* (10.0%). Within the genus *Microtus, M. arvalis* represented the most biomass (5.5% by biomass, 4.2% by numbers), followed by *M. guentheri* (5.4%, 3.4%) and *Microtus* sp. (4.7% by biomass, 4.3% by numbers). Within the genus *Mus*, *Mus* sp. (6.7% by biomass, 10.3% by numbers) and *M. musculus* (7.3%, 9.2%) dominated by biomass, followed by *M. spretus* (2.6%, 4.4%). The genus *Rattus* was mainly represented

by *R. rattus* (11.1%, 1.9%) and *Rattus* sp. (4.3% by biomass, 0.5% by numbers). The biomass of the genus *Apodemus* was dominated by *Apodemus* sp. (5.5% by biomass, 5.6% by numbers) and *A. sylvaticus* (5.4% by biomass, 5.3% by numbers) and the biomass of the genus *Meriones* was composed of *Meriones* sp. (3.5% by biomass, 1.0% of numbers), *M. tristrami* (3.4% by biomass, 1.0% by numbers) and *M. shawi* (1.3% by biomass, 0.2% by numbers). The proportion of birds by prey biomass was 9.0%.

References

Bannikova, A. A., Lebedev, V. S., Kramerov, D. A. & Zaitsev, M. V. Phylogeny and systematics of the *Crocidura suaveolens* species group: corroboration and controversy between nuclear and mitochondrial DNA markers/Phylogénie et systématique du groupe d'espèces *Crocidura suaveolens*: coordination et contradiction des marqueurs nucléaire et mitochondriaux de l'ADN. *Mammalia,* **70**(1-2)*,* 106-119 (2006).

Knitlová, M. & Horáček, I. Late Pleistocene-Holocene paleobiogeography of the genus *Apodemus* in central Europe. *PloS one*, **12**(3), e0173668 (2017).

Kryštufek, B. & Vohralík, V. Mammals of Turkey and Cyprus, Rodentia II: Cricetinae, Murridae, Spalacidae, Calomyscidae, Capromyidae, Hystricidae, Castoridae. *J. Mammal.* **96**, 1-373 (2010).

Table S1 Overall composition of Barn owl diet in a Mediterranean region based on meta-analysis including own pellet collections and published datasets (n = 85 localities). n – number of prey items, number of localities with prey taxa presence in Western (W), central (C), Eastern (E), Levant (L) Mediterranean and total number (T) of localities with prey taxa presence.

Taxa	n	W	C	E	L	T	Mean weight (g)	% by numbers	Total biomass (g)	% by biomass
Aves	16,280	19	13	24	23	79	28.7	8.93	467,236.0	9.01
Erinaceus concolor	4	1	0	2	1	4	360.0	<0.01	1,440.0	0.03
Atelerix algirus	2	2	0	0	0	2	317.0	<0.01	634.0	0.01
Erinaceus roumanicus	1	0	0	1	0	1	335.0	<0.01	335.0	0.01
Hemiechinus auritus	1	0	0	0	1	1	265.0	<0.01	265.0	0.01
Paraechinus aethiopicus	4	0	0	0	1	1	395.0	<0.01	1,580.0	0.03
Talpa europaea	42	1	1	2	0	4	85.0	0.02	3,570.0	0.07
Talpa stankovici	8	0	0	2	0	2	85.0	<0.01	680.0	0.01
Talpa romana	8	0	1	0	0	1	82.0	<0.01	656.0	0.01
Talpa levantis	2	0	0	1	0	1	67.0	<0.01	134.0	<0.01
Crocidura sp.	405	4	3	3	2	12	8.5	0.22	3,442.5	0.07
Crocidura suaveolens	16,024	3	10	24	15	52	7.9	8.79	126,589.6	2.44
Crocidura suaveolens cypria	120	0	0	0	3	3	8.2	0.07	984.0	0.02
Crocidura pachyura	30	1	0	0	0	1	8.0	0.02	240.0	<0.01
Crocidura whittakeri	90	6	0	0	0	6	8.1	0.05	729.0	0.01
Crocidura leucodon	7,365	0	6	12	7	25	9.7	4.04	71,661.5	1.38
Crocidura russula	11,600	12	4	0	2	18	8.8	6.36	102,080.0	1.97
Suncus etruscus	2,628	5	11	18	15	49	1.9	1.44	4,993.2	0.10
Sorex araneus	1,598	1	3	4	0	8	7.3	0.88	11,665.4	0.22
Sorex samniticus	61	0	1	0	0	1	8.4	0.03	512.4	0.01
Sorex minutus	1,146	1	4	3	0	8	3.7	0.63	4,240.2	0.08
Soricinae unidet.	277	0	1	1	4	6	8.0	0.15	2,216.0	0.04
Neomys anomalus	2,334	1	1	5	0	7	12.7	1.28	29,525.1	0.57

Neomys fodiens	8	1	1	0	0	2	17.8	<0.01	142.4	<0.01
Spalax ehrenbergi	204	0	0	0	11	11	137.3	0.11	28,009.2	0.54
Spalax leucodon	4	0	0	0	1	1	198.9	<0.01	795.6	0.02
Oryctolagus cuniculus	52	4	0	1	0	5	94.3	0.03	4,901.0	0.09
Glis glis	14	1	2	3	0	6	94.3	0.01	1,319.5	0.03
Psammomys obesus	7	1	1	0	1	3	212.0	<0.01	1,484.0	0.03
Pachyuromys duprasi	38	3	1	0	0	4	36.5	0.02	1,387.0	0.03
Dryomys nitedula	12	0	0	2	1	3	29.7	0.01	356.4	0.01
Muscardinus avellanarius	279	0	5	4	0	9	20.4	0.15	5,691.6	0.11
Eliomys quercinus	294	7	4	0	0	11	86.0	0.16	25,284.0	0.49
Eliomys melanurus	1	0	0	0	1	1	54.0	<0.01	54.0	<0.01
Elephantulus rozeti	4	2	0	0	0	2	56.0	<0.01	224.0	<0.01
Jaculus jaculus	195	1	1	0	3	5	60.0	0.11	11,700.0	0.23
Jaculus orientalis	23	2	0	0	0	2	134.0	0.01	3,082.0	0.06
Allactaga euphratica	1	0	0	0	1	1	66.3	<0.01	66.3	<0.01
Mesocricetus auratus	67	0	0	0	1	1	93.0	0.04	6,231.0	0.12
Myomimus roachi	7	0	0	2	0	2	46.6	<0.01	326.2	0.01
Cricetulus migratorius	304	0	0	4	9	13	32.8	0.17	9,971.2	0.19
Meriones sp.	1,811	0	0	0	4	4	100.0	0.99	181,100.0	3.49
Meriones tristrami	1,860	0	0	2	13	15	96.0	1.02	178,560.0	3.44
Meriones shawi	374	5	0	0	0	5	185.0	0.21	69,190.0	1.33
Meriones vinogradovi	64	0	0	1	0	1	121.0	0.04	7,744.0	0.15
Meriones persicus	1	0	0	0	1	1	114.5	<0.01	114.5	<0.01
Meriones crassus	285	0	0	0	3	3	65.1	0.16	18,553.5	0.36
Meriones sacramenti	49	0	0	0	1	1	118.0	0.03	5,782.0	0.11
Meriones libycus	19	0	1	0	0	1	101.5	0.01	1,928.5	0.04
Dipodillus maghrebi	966	2	0	0	0	2	20.0	0.53	19,320.0	0.37
Gerbillus sp.	107	6	0	0	0	6	30.0	0.06	3,210.0	0.06
Gerbillus nanus	278	5	0	0	1	6	18.5	0.15	5,143.0	0.10

Gerbillus campestris	1,869	8	0	0	0	8	28.4	1.02	53,079.6	1.02
Gerbillus amoenus	7	0	0	1	0	1	29.0	<0.01	203.0	<0.01
Gerbillus henleyi	36	0	0	0	2	2	31.0	0.02	1,116.0	0.02
Gerbillus dasyurus	10	0	0	0	2	2	30.0	0.01	300.0	0.01
Gerbillus tarabuli	32	3	0	0	0	3	30.0	0.02	960.0	0.02
Gerbillus simoni	2	2	0	0	0	2	26.0	<0.01	52.0	<0.01
Gerbillus allenbyi	341	0	0	0	1	1	28.4	0.19	9,684.4	0.19
Gerbillus gerbillus	225	3	0	0	3	6	32.0	0.12	7,200.0	0.14
Gerbillus pyramidum	307	0	1	0	1	2	28.0	0.17	8,596.0	0.17
Acomys cahirinus minous	17	0	0	2	1	3	43.0	0.01	731.0	0.01
Acomys cf. *cilicicus*	54	0	0	0	1	1	40.2	0.03	2,170.8	0.04
Acomys cahirinus nesiotes	17	0	0	0	2	2	48.3	0.01	821.1	0.02
Acomys dimidiatus	68	0	0	1	3	4	38.8	0.04	2,638.4	0.05
Apodemus sp.	10,216	1	3	8	0	12	28.0	5.60	286,048.0	5.51
Apodemus flavicollis	1,194	0	2	7	3	12	29.7	0.65	35,461.8	0.68
Apodemus epimelas	71	0	0	5	0	5	40.0	0.04	2,840.0	0.05
Apodemus mystacinus	579	0	0	8	5	13	40.4	0.32	23,391.6	0.45
Apodemus sylvaticus	9,725	9	9	14	0	32	28.7	5.33	279,107.5	5.38
Apodemus witherbyi	32	0	0	0	1	1	24.0	0.02	768.0	0.01
Apodemus agrarius	357	0	0	2	0	2	21.5	0.20	7,675.5	0.15
Apodemus uralensis	33	0	0	1	0	1	19.3	0.02	636.9	0.01
Micromys minutus	871	1	1	5	0	7	8.9	0.48	7,751.9	0.15
Mus sp.	18,814	11	2	11	12	36	18.5	10.32	348,059.0	6.71
Mus musculus	16,679	16	11	11	9	47	22.7	9.15	378,613.3	7.30
Mus spretus	7,961	12	0	0	0	12	17.0	4.37	135,337.0	2.61
Mus cf. *macedonicus*	5,248	0	0	11	2	13	16.9	2.88	88,691.2	1.71
Mastomys erythroleucus	9	1	0	0	0	1	37.0	<0.01	333.0	0.01
Lemniscomys barbarus	109	4	0	0	0	4	26.2	0.06	2,855.8	0.06
Rattus sp.	921	8	3	1	5	17	240.0	0.51	221,040.0	4.26

Rattus rattus	3,379	13	8	20	10	51	170.0	1.85	574,430.0	11.07
Rattus norvegicus	260	9	2	3	5	19	260.0	0.14	67,600.0	1.30
Muridae unidet.	126	1	1	3	3	8	25.0	0.07	3,150.0	0.06
Arvicola terrestris	182	0	1	6	1	8	155.0	0.10	28,210.0	0.54
Arvicola sapidus	94	4	0	0	0	4	156.0	0.05	14,664.0	0.28
Clethrionomys glareolus	577	1	4	0	0	5	25.6	0.32	14,771.2	0.28
Microtus sp.	7,866	2	2	2	4	10	31.0	4.31	243,846.0	4.70
Microtus gerbei	15	1	0	0	0	1	20.7	0.01	310.5	0.01
Microtus duodecimcostatus	2,336	5	0	0	0	5	23.0	1.28	53,728.0	1.04
Microtus irani	824	0	0	0	1	1	36.0	0.45	29,664.0	0.57
Microtus socialis	435	0	0	0	3	3	27.9	0.24	12,136.5	0.23
Microtus agrestis	2,096	1	0	0	1	2	39.0	1.15	81,744.0	1.58
Microtus guentheri	6,168	0	0	4	8	12	45.7	3.38	281,877.6	5.43
Microtus levis	809	0	0	7	0	7	38.4	0.44	31,065.6	0.60
Microtus savii	4,910	0	9	0	0	9	20.0	2.69	98,200.0	1.89
Microtus arvalis	7,738	1	1	4	0	6	36.9	4.24	285,532.2	5.50
Chionomys gud	14	1	0	1	0	2	40.5	0.01	567.0	0.01
Microtus subterraneus	182	0	0	3	0	3	18.9	0.10	3,439.8	0.07
Microtus thomasi	833	0	0	5	0	5	30.0	0.46	24,990.0	0.48
Tatera indica	5	0	0	0	1	1	147.0	<0.01	735.0	0.01
Mustela nivalis	3	1	0	2	0	3	92.0	<0.01	276.0	0.01
Chiroptera unidet.	164	5	4	0	0	9	10.0	0.09	1,640.0	0.03
Eptesicus serotinus	2	0	0	0	1	1	25.0	<0.01	50.0	<0.01
Miniopterus schreibersii	2	0	1	0	1	2	12.5	<0.01	25.0	<0.01
Myotis blythii	5	0	0	1	2	3	21.5	<0.01	107.5	<0.01
Myotis emarginatus	3	0	0	0	1	1	8.1	<0.01	24.3	<0.01
Myotis capaccinii	1	0	0	1	0	1	9.7	<0.01	9.7	<0.01
Myotis myotis	7	1	0	2	2	5	24.0	<0.01	168.0	<0.01
Myotis nattereri	6	0	0	0	2	2	8.5	<0.01	51.0	<0.01

Nycteris thebaica	1	0	0	0	1	1	8.0	<0.01	8.0	<0.01
Hypsugo savii	1	0	1	0	0	1	6.5	<0.01	6.5	<0.01
Pipistrellus sp.	9	2	0	2	0	4	6.5	<0.01	58.5	<0.01
Pipistrellus kuhlii	99	0	0	1	5	6	7.5	0.05	742.5	0.01
Pipistrellus nathusii	4	0	0	1	0	1	7.0	<0.01	28.0	<0.01
Pipistrellus pipistrellus	11	0	1	1	1	3	4.6	0.01	50.6	<0.01
Rousettus aegyptiacus	7	0	0	0	3	3	135.0	<0.01	945.0	0.02
Rhinolophus ferrumequinum	4	0	0	0	3	3	24.0	<0.01	96.0	<0.01
Otonycteris hemprichii	1	0	0	0	1	1	23.0	<0.01	23.0	<0.01
Tadarida teniotis	1	1	0	0	0	1	32.0	<0.01	32.0	<0.01
Plecotus kolombatovici	1	0	0	1	0	1	13.3	<0.01	13.3	<0.01
Total/mean	182,343						61.5	100.00	5,188,258.9	100.00
