# Supplementary material for: Large-scale spatial patterns of small-mammal communities in the Mediterranean region revealed by Barn owl diet
Source: Sci Rep. 2021 Mar 2;11:4985. doi: 10.1038/s41598-021-84683-y (PMC7970837; doi:10.1038/s41598-021-84683-y)

Supplementary Material 2

Inferring large-scale spatial patterns of small-mammal communities in the Mediterranean region revealed by Barn owl diet

Jan Riegert, Jiří Šindelář, Markéta Zárybnická & Ivan Horáček

Figure S1 Diversity of small-mammals in the diet of Barn owl along longitude within Mediterranean with delimited subregions for a) mainland and b) island localities.

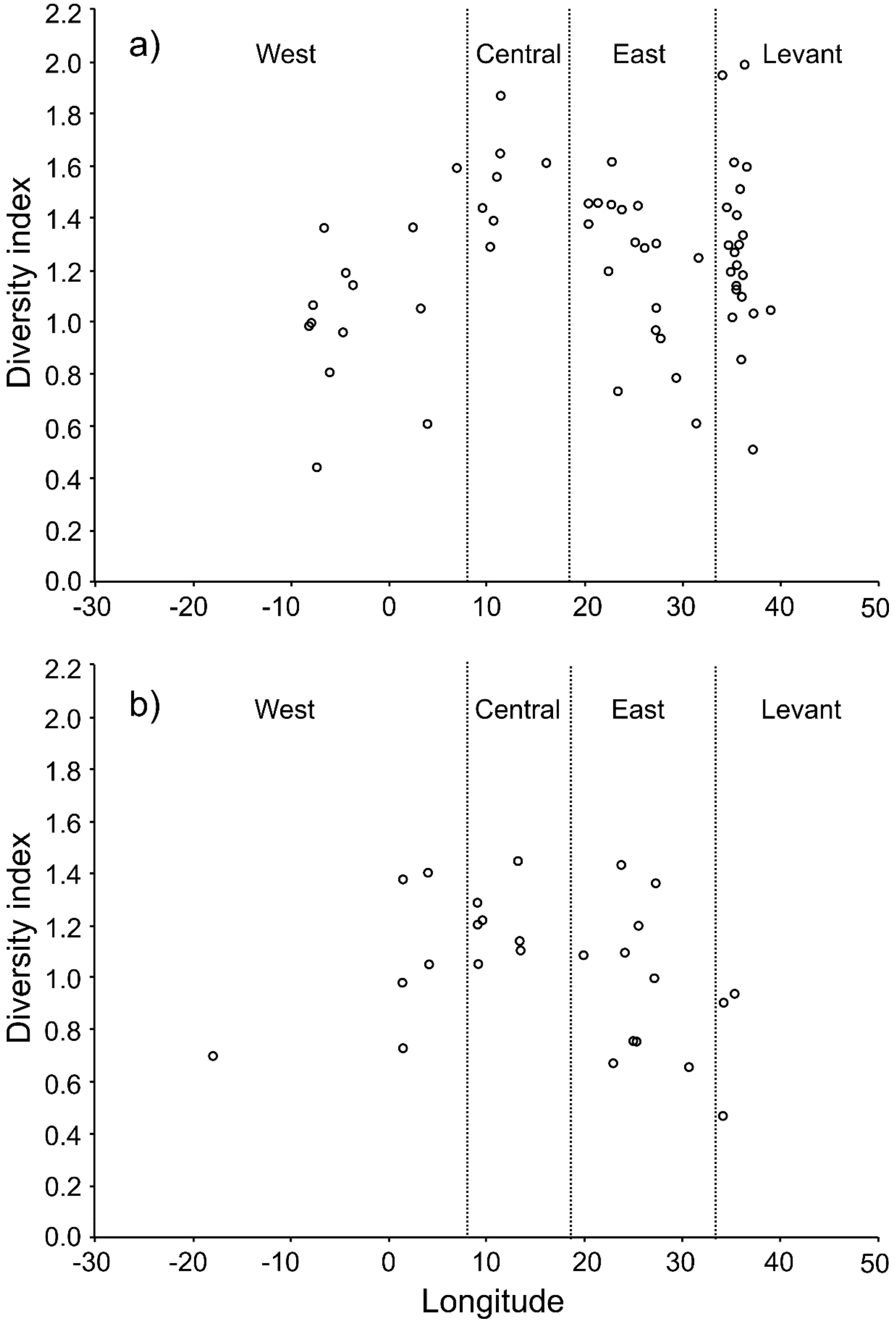

Figure S2 Diversity of small-mammals in the diet of Barn owl along latitude within Mediterranean for mainland localities in longitudinal subregions.

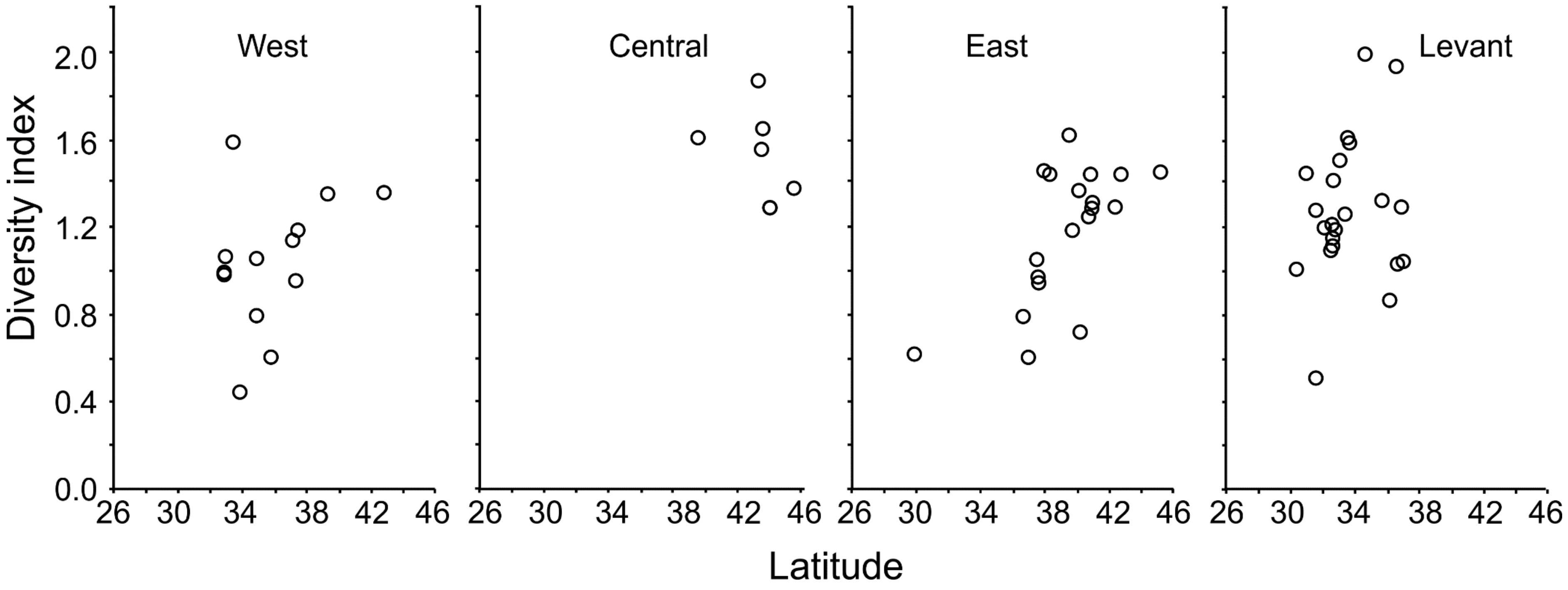

Supplement: Supplementary file 2 — Supplementary Information 2. [file 41598_2021_84683_MOESM2_ESM.pdf]
