# Supplementary material for: Large-scale spatial patterns of small-mammal communities in the Mediterranean region revealed by Barn owl diet
Source: Sci Rep. 2021 Mar 2;11:4985. doi: 10.1038/s41598-021-84683-y (PMC7970837; doi:10.1038/s41598-021-84683-y)
Supplement: Supplementary file 3 — Supplementary Information 3. [file 41598_2021_84683_MOESM3_ESM.pdf]

Supplementary Material 3

Inferring large-scale spatial patterns of small-mammal communities in the Mediterranean region revealed by Barn owl diet

Jan Riegert, Jiří Šindelář, Markéta Zárybnická & Ivan Horáček

Table S2 The results of multi-model inference comparison for GLMM models containing various combinations of variables based on AIC values for Barn owl diet diversity and mean prey weight for all localities (n = 85). Legend for variable codes: Lat – latitude, Lon – longitude, Isl – island/mainland, LmR – land modification range, LmM – mean land modification.

Dependent variable	Model	df	logLik	AICc	Delta	Weight
**Diet diversity**	Isl+Lat	6	-23.90	60.90	0.00	0.45
	Isl	5	-26.04	62.86	1.96	0.17
	Null model	4	-27.40	63.30	2.40	0.14
	Lat	5	-26.54	63.87	2.97	0.10
	Isl+LmR	6	-26.37	65.84	4.94	0.04
	Isl+LmM	6	-26.50	66.11	5.21	0.03
	LmR	5	-27.74	66.25	5.35	0.03
	LmM	5	-27.96	66.70	5.80	0.02
	Isl+LmM+LmR	7	-26.83	69.16	8.25	0.01
	LmM+LmR	6	-28.29	69.68	8.78	0.01
	Isl+Lat+Lon	7	-27.71	70.90	10.00	<0.01
	Isl+LmM+Lat+Lon	8	-28.21	74.36	13.46	<0.01
	Lon	5	-31.84	74.45	13.55	<0.01
	Isl+Lon	6	-30.84	74.78	13.88	<0.01
	Isl+LmM+LmR+Lat+Lon	9	-28.34	77.15	16.25	<0.01
	LmM+LmR+Lat+Lon	8	-30.32	78.59	17.69	<0.01
	LmM+LmR+Lon	7	-32.74	80.98	20.08	<0.01
**Mean prey weight (g)**	Isl+LmM+LmR+Lat	8	-363.73	745.41	<0.01	0.82
	Isl+LmM+LmR+Lat+Lon	9	-364.49	749.44	4.03	0.11
	Isl+LmM+Lat	7	-368.54	752.58	7.16	0.02
	Isl+LmR+Lat	7	-368.63	752.76	7.34	0.02
	LmM+LmR+Lat+Lon	8	-367.61	753.16	7.75	0.02
	Isl+LmR+Lat+Lon	8	-369.33	756.61	11.19	<0.01
	LmM+LmR	6	-372.40	757.90	12.48	<0.01
	Isl+LmM	6	-373.48	760.06	14.65	<0.01
	Isl+Lat	6	-373.50	760.11	14.69	<0.01
	LmM+LmR+Lon	7	-372.96	761.41	16.00	<0.01
	LmM	5	-376.52	763.82	18.41	<0.01
	Isl+Lat+Lon	7	-374.24	763.97	18.55	<0.01
	LmR	5	-377.11	765.01	19.59	<0.01
	Lat	5	-377.37	765.52	20.10	<0.01
	Isl	5	-377.89	766.55	21.14	<0.01
	Null model	4	-381.32	771.15	25.74	<0.01
	Lon	5	-382.08	774.94	29.53	<0.01

Table S3 The results of multi-model inference comparison for GLMM models containing various combination of variables based on AIC values for Barn owl diet diversity and mean prey weight on islands (n = 25 localities). Legend for variable codes: Lat – latitude, Lon – longitude, IslA – area of island, IslD – distance of island from mainland, LmR – land modification range, LmM – mean land modification.

Dependent variable	Model	df	logLik	AICc	Delta	Weight
**Diet diversity**	Lat	3	2.09	2.96	0.00	0.75
	IslA+IslD+Lat	5	2.56	8.05	5.08	0.06
	Null model	2	-1.94	8.43	5.47	0.05
	IslD	3	-1.17	9.49	6.52	0.03
	IslA	3	-1.37	9.89	6.93	0.02
	LmM	3	-1.69	10.53	7.57	0.02
	Lon	3	-1.70	10.54	7.58	0.02
	LmR	3	-1.93	11.00	8.04	0.01
	IslA+IslD+Lat+Lon	6	2.74	11.18	8.22	0.01
	LmM+LmR+Lat+Lon	6	2.42	11.84	8.88	0.01
	IslA+IslD	4	-0.94	11.89	8.92	0.01
	LmM+LmR	4	-1.63	13.27	10.31	<0.01
	IslA+IslD+LmR	5	-0.94	15.04	12.08	<0.01
	IslA+IslD+LmR+Lat+Lon	7	2.75	15.09	12.13	<0.01
	IslA+LmM+LmR+Lat+Lon	7	2.72	15.15	12.19	<0.01
	LmM+LmR+Lon	5	-1.42	16.01	13.05	<0.01
	IslA+IslD+LmM+LmR+Lat+Lon	8	2.75	19.50	16.54	<0.01
**Mean prey weight (g)**	LmM+LmR+Lat	5	-106.87	226.9	0.00	0.23
	IslA+IslD	4	-108.65	227.31	0.41	0.19
	IslA	3	-110.13	227.41	0.51	0.18
	LmM+LmR	4	-109.09	228.18	1.28	0.12
	LmR	3	-111.34	229.82	2.92	0.05
	LmM+LmR+Lat+Lon	6	-106.63	229.92	3.02	0.05
	IslA+IslD+LmM	5	-108.50	230.15	3.25	0.05
	IslA+IslD+Lon	5	-108.56	230.27	3.37	0.04
	Null model	2	-113.48	231.5	4.60	0.02
	LmM	3	-112.50	232.14	5.24	0.02
	IslA+LmM+LmR+Lat+Lon	7	-106.19	232.97	6.07	0.01
	IslA+IslD+Lat+Lon	6	-108.23	233.13	6.23	0.01
	Lon	3	-113.11	233.36	6.46	0.01
	IslD	3	-113.37	233.88	6.98	0.01
	Lat	3	-113.42	233.99	7.09	0.01
	IslA+IslD+LmM+LmR+Lat+Lon	8	-104.63	234.26	7.37	0.01
	IslA+IslD+LmR+Lat+Lon	7	-107.23	235.04	8.14	<0.01

Table S4 The results of multi-model inference comparison for GLMM models containing various combinations of variables based on AIC values for Barn owl diet diversity for all localities within the Mediterranean subregions. Legend for variable codes: Lat – latitude, Lon – longitude, Isl – island/mainland, LmR – land modification range, LmM – mean land modification.

Subregion	Model	df	logLik	AICc	Delta	Weight
**West**	LmM+LmR+Lat+Lon	6	0.91	-73.82	0.00	0.50
	Isl+LmM+LmR+Lat+Lon	6	0.91	-73.82	0.00	0.50
	Null model	2	-0.85	9.69	83.51	<0.01
	Isl	2	-0.85	9.69	83.51	<0.01
	Lat	3	0.48	17.04	90.86	<0.01
	Lon	3	0.43	17.13	90.95	<0.01
	Isl+Lon	3	0.43	17.13	90.95	<0.01
	LmR	3	-0.29	18.58	92.40	<0.01
	LmM	3	-0.78	19.56	93.38	<0.01
	Isl+LmM	3	-0.78	19.56	93.38	<0.01
	Isl+Lat+Lon	4	0.60	46.8	120.61	<0.01
	LmM+LmR	4	-0.14	48.29	122.11	<0.01
	Isl+LmM+LmR	4	-0.14	48.29	122.11	<0.01
	LmM+LmR+Lat	5	0.81	51.23	125.05	<0.01
	Isl+LmM+Lat+Lon	5	0.79	52.35	126.17	<0.01
**Central**	Null model	2	4.75	-2.49	0.00	0.45
	Isl	2	4.75	-2.49	0.00	0.45
	LmR	3	5.28	3.45	5.94	0.02
	LmM	3	4.89	4.22	6.72	0.02
	Isl+LmM	3	4.89	4.22	6.72	0.02
	Lon	3	4.82	4.35	6.84	0.01
	Isl+Lon	3	4.82	4.35	6.84	0.01
	Lat	3	4.78	4.43	6.93	0.01
	LmM+LmR	4	5.31	17.37	19.86	<0.01
	Isl+LmM+LmR	4	5.31	17.37	19.86	<0.01
	Isl+Lat+Lon	4	4.93	18.13	20.62	<0.01
	Isl+LmM+Lat+Lon	5	7.26	55.48	57.97	<0.01
	LmM+LmR+Lat	5	7.21	55.59	58.08	<0.01
	LmM+LmR+Lat+Lon	6	7.67	61.21	63.70	<0.01
	Isl+LmM+LmR+Lat+Lon	6	7.67	63.24	65.73	<0.01
**East**	Null model	2	-0.47	6.94	0.00	0.38
	Isl	2	-0.47	6.94	0.00	0.38
	Lat	3	-0.03	10.87	3.93	0.05
	Lon	3	-0.40	11.59	4.66	0.04
	Isl+Lon	3	-0.40	11.59	4.66	0.04
	LmM	3	-0.42	11.63	4.70	0.04
	Isl+LmM	3	-0.42	11.63	4.70	0.04

	LmR	3	-0.47	11.73	4.80	0.03
	Isl+Lat+Lon	4	0.27	17.46	10.52	<0.01
	LmM+LmR	4	-0.40	18.80	11.87	<0.01
	Isl+LmM+LmR	4	-0.40	18.80	11.87	<0.01
	Isl+LmM+Lat+Lon	5	0.52	28.96	22.02	<0.01
	LmM+LmR+Lat	5	0.29	29.43	22.49	<0.01
	LmM+LmR+Lat+Lon	6	0.65	52.70	45.77	<0.01
	Isl+LmM+LmR+Lat+Lon	6	0.65	52.70	45.77	<0.01
**Levant**	LmM+LmR	4	100.21	-212.42	0.00	0.20
	LmM+LmR+Lat	4	100.21	-212.42	0.00	0.20
	LmM+LmR+Lat+Lon	4	100.21	-212.42	0.00	0.20
	Isl+LmM+LmR+Lat+Lon	4	100.21	-212.42	0.00	0.20
	Isl+LmM+LmR	4	100.21	-212.42	0.00	0.20
	Isl+LmM+Lat+Lon	4	90.11	-192.21	20.21	<0.01
	Isl+Lat+Lon	4	87.73	-187.46	24.96	<0.01
	LmR	3	5.88	-29.75	182.67	<0.01
	Lon	3	1.98	-21.96	190.46	<0.01
	Isl+Lon	3	1.98	-21.96	190.46	<0.01
	LmM	3	1.54	-21.08	191.35	<0.01
	Isl+LmM	3	1.54	-21.08	191.35	<0.01
	Lat	3	0.89	-19.77	192.65	<0.01
	Null model	2	0.43	-15.22	197.20	<0.01
	Isl	2	0.43	-13.10	199.32	<0.01

Table S5 The results of multi-model inference comparison for GLMM models containing various combinations of variables based on AIC values for Barn owl mean prey weight for all localities within the Mediterranean subregions. Legend for variable codes: Lat – latitude, Lon – longitude, Isl – island/mainland, LmR – land modification range, LmM – mean land modification.

Subregion	Model	df	logLik	AICc	Delta	Weight
**West**	LmM+LmR+Lat+Lon	6	-19.51	-32.97	0.00	0.50
	Isl+LmM+LmR+Lat+Lon	6	-19.51	-32.97	0.00	0.50
	Null model	2	-25.24	58.48	91.45	<0.01
	Isl	2	-25.24	58.48	91.45	<0.01
	LmR	3	-24.75	67.50	100.47	<0.01
	Lon	3	-24.76	67.52	100.50	<0.01
	Isl+Lon	3	-24.76	67.52	100.50	<0.01
	Lat	3	-24.77	67.54	100.51	<0.01
	LmM	3	-25.08	68.16	101.13	<0.01
	Isl+LmM	3	-25.08	68.16	101.13	<0.01
	LmM+LmR	4	-22.17	92.34	125.31	<0.01
	Isl+LmM+LmR	4	-22.17	92.34	125.31	<0.01
	Isl+Lat+Lon	4	-24.75	97.50	130.47	<0.01
	LmM+LmR+Lat	5	-22.17	101.21	134.18	<0.01
	Isl+LmM+Lat+Lon	5	-23.15	105.61	138.58	<0.01
**Central**	Null model	2	-24.02	55.03	0.00	0.43
	Isl	2	-24.02	55.03	0.00	0.43
	Lat	3	-23.21	60.42	5.38	0.03
	Lon	3	-23.34	60.68	5.64	0.03
	Isl+Lon	3	-23.34	60.68	5.64	0.03
	LmM	3	-23.54	61.07	6.04	0.02
	Isl+LmM	3	-23.54	61.07	6.04	0.02
	LmR	3	-23.68	61.36	6.33	0.02
	Isl+Lat+Lon	4	-23.18	74.36	19.32	<0.01
	LmM+LmR	4	-23.36	74.71	19.68	<0.01
	Isl+LmM+LmR	4	-23.36	74.71	19.68	<0.01
	LmM+LmR+Lat	5	-18.08	106.16	51.13	<0.01
	Isl+LmM+Lat+Lon	5	-20.63	111.25	56.22	<0.01
	LmM+LmR+Lat+Lon	6	-17.35	115.64	60.61	<0.01
	Isl+LmM+LmR+Lat+Lon	6	-17.35	117.53	62.50	<0.01
**East**	LmM	3	-40.48	91.76	0.00	0.22
	Isl+LmM	3	-40.48	91.76	0.00	0.22
	Null model	2	-43.27	92.54	0.78	0.15
	Isl	2	-43.27	92.54	0.78	0.15
	LmR	3	-41.03	92.86	1.10	0.13
	LmM+LmR	4	-38.45	94.91	3.15	0.05
	Isl+LmM+LmR	4	-38.45	94.91	3.15	0.05

	Lon	3	-43.03	96.86	5.10	0.02
	Isl+Lon	3	-43.03	96.86	5.10	0.02
	Lat	3	-43.27	97.33	5.57	0.01
	Isl+Lat+Lon	4	-43.01	104.03	12.27	<0.01
	LmM+LmR+Lat	5	-38.45	106.91	15.15	<0.01
	Isl+LmM+Lat+Lon	5	-39.34	108.67	16.91	<0.01
	LmM+LmR+Lat+Lon	6	-36.46	126.92	35.16	<0.01
	Isl+LmM+LmR+Lat+Lon	6	-36.46	126.92	35.16	<0.01
**Levant**	LmM+LmR	4	90.88	-193.75	0.00	0.20
	LmM+LmR+Lat	4	90.88	-193.75	0.00	0.20
	LmM+LmR+Lat+Lon	4	90.88	-193.75	0.00	0.20
	Isl+LmM+LmR+Lat+Lon	4	90.88	-193.75	0.00	0.20
	Isl+LmM+LmR	4	90.88	-193.75	0.00	0.20
	Isl+LmM+Lat+Lon	4	79.73	-171.46	22.29	<0.01
	Isl+Lat+Lon	4	76.14	-164.28	29.47	<0.01
	LmR	3	-8.64	-0.72	193.03	<0.01
	LmM	3	-9.32	0.64	194.39	<0.01
	Isl+LmM	3	-9.32	0.64	194.39	<0.01
	Lon	3	-10.65	3.29	197.04	<0.01
	Isl+Lon	3	-10.65	3.29	197.04	<0.01
	Lat	3	-11.29	4.58	198.33	<0.01
	Null model	2	-11.42	5.67	199.42	<0.01
	Isl	2	-11.42	6.78	200.53	<0.01

Table S6 The results of multi-model inference comparison for GLMM models containing various combinations of variables based on AIC values for Barn owl diet diversity for mainland localities within the Mediterranean subregions. Legend for variable codes: Lat – latitude, Lon – longitude, LmR – land modification range, LmM – mean land modification.

Subregion	Model	df	logLik	AICc	Delta	Weight
**West**	LmM	3	-0.91	10.48	0.00	0.29
	Null model	2	-2.65	10.51	0.02	0.28
	Lat	3	-1.74	12.14	1.66	0.13
	Lon	3	-1.85	12.36	1.88	0.11
	LmR	3	-2.53	13.73	3.25	0.06
	LmM+Lon	4	-0.82	14.65	4.16	0.04
	LmM+Lat	4	-0.83	14.66	4.18	0.04
	LmM+LmR	4	-0.91	14.81	4.33	0.03
	LmR+Lat	4	-1.74	16.48	5.99	0.01
	LmR+Lon	4	-1.84	16.68	6.19	0.01
	LmM+LmR+Lat	5	-0.82	20.21	9.72	<0.01
	LmR+Lat+Lon	5	-1.09	20.74	10.26	<0.01
	LmM+LmR+Lat+Lon	6	-0.64	27.29	16.80	<0.01
**Central**	Null model	2	2.13	2.74	0.00	0.85
	LmM	3	2.72	8.57	5.83	0.05
	Lon	3	2.57	8.85	6.11	0.04
	LmR	3	2.55	8.90	6.16	0.04
	Lat	3	2.13	9.73	6.99	0.03
	LmM+Lat	4	3.77	20.46	17.73	<0.01
	LmM+LmR	4	3.33	21.34	18.60	<0.01
	LmM+Lon	4	3.32	21.36	18.63	<0.01
	LmR+Lon	4	2.75	22.50	19.77	<0.01
	LmR+Lat	4	2.65	22.70	19.96	<0.01
	LmM+LmR+Lat	5	3.81	62.38	59.64	<0.01
	LmR+Lat+Lon	5	2.78	64.44	61.71	<0.01
	LmM+LmR+Lat+Lon	6	6.13	66.87	64.13	<0.01
**East**	Lon	3	3.10	1.52	0.00	0.60
	LmR+Lon	4	3.25	4.58	3.06	0.13
	LmM+Lon	4	3.21	4.65	3.13	0.13
	LmR+Lat+Lon	5	4.59	5.83	4.31	0.07
	Lat	3	0.49	6.73	5.21	0.04
	LmR+Lat	4	0.71	9.65	8.13	0.01
	LmM++Lat	4	0.71	9.66	8.14	0.01
	LmM+LmR+Lat+Lon	6	4.60	10.45	8.93	0.01
	LmM++LmR+Lat	5	1.06	12.88	11.36	<0.01
	Null+model	2	-4.49	13.78	12.25	<0.01
	LmR+	3	-4.48	16.67	15.15	<0.01
	LmM+	3	-4.48	16.68	15.16	<0.01

	LmM+LmR	4	-4.47	20.01	18.49	<0.01
**Levant**	Lon	3	-4.67	16.68	0.00	0.28
	Null model	2	-6.42	17.47	0.80	0.19
	LmR+Lat+Lon	5	-2.47	18.70	2.02	0.10
	LmM+Lon	4	-4.24	18.83	2.16	0.10
	Lat	3	-6.03	19.39	2.71	0.07
	LmR+Lon	4	-4.56	19.47	2.79	0.07
	LmR	3	-6.14	19.61	2.93	0.07
	LmM	3	-6.34	20.02	3.34	0.05
	LmR+Lat	4	-5.91	22.17	5.49	0.02
	LmM+LmR	4	-6.01	22.37	5.70	0.02
	LmM+Lat	4	-6.02	22.39	5.72	0.02
	LmM+LmR+Lat+Lon	6	-2.40	22.41	5.73	0.02
	LmM+LmR+Lat	5	-5.87	25.50	8.82	<0.01

Table S7 The results of multi-model inference comparison for GLMM models containing various combinations of variables based on AIC values for Barn owl mean prey weight for mainland localities within the Mediterranean subregions. Legend for variable codes: Lat – latitude, Lon – longitude, LmR – land modification range, LmM – mean land modification.

Subregion	Model	df	logLik	AICc	Delta	Weight
**West**	Lon	3	-65.13	138.93	0.00	0.40
	Null model	2	-67.35	139.91	0.98	0.25
	LmM	3	-66.72	142.10	3.17	0.08
	LmR	3	-66.85	142.37	3.44	0.07
	Lat	3	-67.27	143.20	4.27	0.05
	LmM+Lon	4	-65.13	143.26	4.32	0.05
	LmR+Lon	4	-65.13	143.26	4.33	0.05
	LmM+Lat	4	-65.69	144.37	5.44	0.03
	LmM+LmR	4	-66.47	145.93	7.00	0.01
	LmR+Lat	4	-66.47	145.94	7.01	0.01
	LmR+Lat+Lon	5	-64.43	147.42	8.49	0.01
	LmM+LmR+Lat	5	-65.00	148.57	9.64	<0.01
	LmM+LmR+Lat+Lon	6	-63.96	153.91	14.98	<0.01
**Central**	Lat	3	-19.35	52.70	0.00	0.79
	LmR+Lat	4	-13.80	55.61	2.91	0.18
	Null model	2	-26.73	60.46	7.76	0.02
	LmR	3	-24.04	62.07	9.37	0.01
	LmM	3	-25.23	64.47	11.77	<0.01
	LmM+Lat	4	-18.65	65.30	12.60	<0.01
	Lon	3	-26.61	67.21	14.51	<0.01
	LmM+LmR	4	-20.72	69.44	16.74	<0.01
	LmR+Lon	4	-23.87	75.75	23.05	<0.01
	LmM+Lon	4	-25.10	78.20	25.50	<0.01
	LmR+Lat+Lon	5	-11.83	93.66	40.96	<0.01
	LmM+LmR+Lat	5	-13.8	97.61	44.91	<0.01
	LmM+LmR+Lat+Lon	6	-10.37	101.32	48.62	<0.01
**East**	Null model	2	-71.11	147.02	0.00	0.27
	LmM	3	-70.30	148.31	1.29	0.14
	Lat	3	-70.36	148.44	1.43	0.13
	LmR	3	-70.50	148.72	1.70	0.12
	LmM+LmR	4	-69.22	149.51	2.50	0.08
	Lon	3	-71.03	149.77	2.76	0.07
	LmM+Lat	4	-69.70	150.47	3.45	0.05
	LmR+Lat	4	-69.87	150.82	3.80	0.04
	LmM+Lon	4	-70.27	151.61	4.59	0.03
	LmR+Lon	4	-70.38	151.85	4.83	0.02
	LmR+Lat+Lon	5	-68.58	152.17	5.15	0.02
	LmM+LmR+Lat	5	-68.79	152.58	5.56	0.02

	LmM+LmR+Lat+Lon	6	-67.96	155.55	8.53	<0.01
**Levant**	Lat	3	-85.04	177.42	0.00	0.37
	LmM+Lat	4	-84.05	178.45	1.03	0.22
	LmR+Lat	4	-84.81	179.97	2.55	0.10
	Null model	2	-87.95	180.53	3.11	0.08
	LmR+Lat+Lon	5	-83.83	181.41	3.99	0.05
	LmR	3	-87.05	181.43	4.01	0.05
	LmM+LmR+Lat	5	-83.99	181.73	4.31	0.04
	LmM	3	-87.87	183.07	5.65	0.02
	Lon	3	-87.92	183.17	5.75	0.02
	LmM+LmR+Lat+Lon	6	-82.98	183.55	6.13	0.02
	LmM+LmR	4	-87.02	184.39	6.97	0.01
	LmR+Lon	4	-87.05	184.45	7.03	0.01
	LmM++Lon	4	-87.85	186.06	8.64	<0.01

Table S8 The effect of latitude, longitude, island/mainland location, mean (mean LM) and range (range LM) of land modification on Barn owl diet diversity and mean prey weight for mainland and all localities regarding to four main subregions within the Mediterranean (GLMM analyses). Significant and marginally significant relationships are in bold.

**Dependent variable**	**Localities**	**Subregion**	**Independent variable**	**Estimate**	**S.E.**	**z**	**P**
**Diet diversity**	All (n = 85)	West	Intercept	-8.89	44.92	0.03	0.976
			Latitude	0.28	1.17	0.04	0.971
			Longitude	-0.12	0.64	0.03	0.977
			Island	-0.01	0.07	0.15	0.881
			Range LM	-0.58	2.87	0.03	0.975
			Mean LM	-0.56	6.31	0.01	0.989
		Central	Intercept	1.19	0.18	5.17	**<0.001**
			Latitude	-0.01	0.03	0.17	0.864
			Longitude	0.01	0.03	0.26	0.797
			Island	-0.34	0.10	3.11	**0.002**
			Range LM	0.76	0.84	0.69	0.491
			Mean LM	0.12	0.26	0.35	0.728
		East	Intercept	0.90	0.67	1.17	0.243
			Latitude	0.04	0.05	0.70	0.483
			Longitude	0.02	0.05	0.28	0.777
			Island	-0.01	0.06	0.16	0.873
			Range LM	0.09	1.43	0.05	0.960
			Mean LM	0.26	0.92	0.24	0.813
		Levant	Intercept	-1.90	5.29	0.07	0.947
			Latitude	0.89	1.48	0.09	0.926
			Longitude	0.69	0.48	0.54	0.587
			Island	-0.71	0.36	1.95	**0.052**
			Range LM	5.45	1.28	0.81	0.416
			Mean LM	-1.48	6.26	0.05	0.964
**Mean prey weight (g)**	All (n = 85)	West	Intercept	1716.25	1351.36	0.20	0.845
			Latitude	-41.92	35.32	0.18	0.855
			Longitude	23.02	19.30	0.18	0.854
			Island	-0.69	7.65	0.08	0.933
			Range LM	132.69	86.45	0.24	0.813
			Mean LM	-407.60	189.76	0.33	0.740
		Central	Intercept	28.51	17.67	1.39	0.164
			Latitude	1.73	1.53	0.86	0.390
			Longitude	-1.54	1.50	0.78	0.434
			Island	-0.32	1.97	0.15	0.883
			Range LM	-37.30	52.61	0.54	0.589

			Mean LM	-12.88	15.04	0.65	0.514
		East	Intercept	65.43	99.11	0.62	0.539
			Latitude	0.29	5.94	0.04	0.968
			Longitude	-3.18	5.15	0.51	0.609
			Island	25.99	10.22	2.46	0.014
			Range LM	243.71	126.01	1.61	0.109
			Mean LM	-185.75	78.74	1.96	**0.050**
		Levant	Intercept	59.33	606.28	0.02	0.987
			Latitude	-39.60	86.83	0.07	0.943
			Longitude	-40.18	32.43	0.47	0.640
			Island	1.51	5.60	0.26	0.794
			Range LM	-203.42	161.97	0.24	0.810
			Mean LM	186.23	233.89	0.15	0.879
**Diet diversity**	Mainland (n = 60)	West	Intercept	1.04	0.84	1.17	0.242
			Latitude	0.03	0.03	0.89	0.376
			Longitude	0.02	0.02	0.84	0.400
			Range LM	0.17	1.00	0.15	0.882
			Mean LM	-1.09	0.65	1.49	0.136
		Central	Intercept	1.53	0.22	5.47	**<0.001**
			Latitude	0.00	0.02	0.05	0.958
			Longitude	0.03	0.04	0.63	0.532
			Range LM	0.36	0.46	0.61	0.544
			Mean LM	-0.36	0.38	0.73	0.467
		East	Intercept	2.50	1.30	1.89	**0.059**
			Latitude	0.05	0.03	1.70	**0.090**
			Longitude	-0.07	0.02	3.88	**<0.001**
			Range LM	0.28	0.50	0.52	0.603
			Mean LM	-0.14	0.47	0.27	0.788
		Levant	Intercept	3.60	2.95	1.19	0.235
			Latitude	0.05	0.05	1.12	0.262
			Longitude	-0.14	0.07	1.77	**0.077**
			Range LM	0.08	0.58	0.13	0.896
			Mean LM	0.23	0.42	0.53	0.596
**Mean prey weight (g)**	Mainland (n = 60)	West	Intercept	67.10	92.90	0.67	0.501
			Latitude	-3.85	5.37	0.65	0.519
			Longitude	4.53	2.25	1.79	**0.074**
			Range LM	87.70	148.67	0.53	0.597
			Mean LM	-90.75	118.61	0.70	0.487
		Central	Intercept	148.67	27.32	4.65	**<0.001**
			Latitude	-2.92	0.52	4.42	**<0.001**
			Longitude	-1.02	2.46	0.32	0.752
			Range LM	-24.85	8.64	2.25	**0.024**

		Mean LM	-15.79	28.14	0.49	0.623
	East	Intercept	27.67	39.68	0.67	0.503
		Latitude	-1.15	1.03	1.03	0.304
		Longitude	-0.55	1.09	0.47	0.637
		Range LM	32.32	29.18	1.02	0.307
		Mean LM	32.05	25.48	1.16	0.246
	Levant	Intercept	123.52	69.29	1.72	**0.085**
		Latitude	-3.46	1.44	2.27	**0.023**
		Longitude	2.65	3.35	0.75	0.453
		Range LM	-12.41	22.49	0.52	0.602
		Mean LM	18.16	16.10	1.06	0.289

Table S9 Results of multivariate CCA analysis on the effect of island/mainland, latitude, longitude, mean land modification, range of land modification and presence/absence of main habitats on representation of mammalian taxa in Barn owl diet within the Mediterranean region. Indicated are also correlation coefficients with the first and second ordination axis. I. and II. ordination axis together explained 65.9% of variability. Significant effects are in bold.

Variable	Axis 1	Axis 2	% of explained variability	Contribution (%)	pseudo-F	P
**Latitude**	-0.68	0.06	8.6	28.5	7.6	**0.002**
**Desert**	-0.56	-0.19	5.3	17.8	5.0	**0.002**
**Island/mainland**	0.23	-0.50	4.4	14.5	4.2	**0.002**
**Longitude**	0.00	-0.64	3.1	10.2	3.0	**0.010**
**Range of land modification**	-0.24	0.31	1.9	6.4	1.9	**0.016**
**Mean land modification**	-0.07	-0.43	1.5	4.9	1.5	0.121
**Bush**	-0.02	-0.27	1.4	4.5	1.4	0.192
**Wetland**	0.09	0.06	1.1	3.6	1.1	0.292
**Agriculture land**	0.21	0.21	1.2	3.9	1.2	0.220
**Forest**	0.29	-0.07	0.9	3.1	0.9	0.532
**Urban**	-0.16	0.07	0.8	2.6	0.8	0.704
