# Supplementary material for: Large-scale spatial patterns of small-mammal communities in the Mediterranean region revealed by Barn owl diet
Source: Sci Rep. 2021 Mar 2;11:4985. doi: 10.1038/s41598-021-84683-y (PMC7970837; doi:10.1038/s41598-021-84683-y)
Supplement: Supplementary file 4 — Supplementary Information 4. [file 41598_2021_84683_MOESM4_ESM.pdf]

Supplementary Material 4

Inferring large-scale spatial patterns of small-mammal communities in the Mediterranean region revealed by Barn owl diet

Jan Riegert, Jiří Šindelář, Markéta Zárybnická & Ivan Horáček

Table S10. Study localities based on literary collections and pellet collections (own data) included in meta-analysis of Barn owl diet. Main geographical and dietary composition characteristics including presence/absence (0/1) of main habitats. Legend: W - West, C - Central, E - East, L - Levant, N – latitude, E – longitude, Diversity index – Shannon diversity index.

ID	Country	Locality	N	E	Subregion	Island area (km^2)	Precipitations (mm)	Number of collection sites	Number of years	Number of mammal inds.	Urban	Agricultural land	Desert	Forest	Bush	Wetland	Number of species	Mean prey mass (g)	Total mammal mass (g)	Diversity index	Reference
1	Algeria	Ain El-Hadjel	35.69	3.91	W		516	1	1	466	0	1	0	0	0	0	13	164.41	76,617	0.61	Sekour et al. (2014)
2	Algeria	Djelfa	34.75	3.23	W		366	1	1	552	0	1	0	1	0	0	16	125.69	69,382	1.05	Sekour et al. (2014)
3	Algeria	El Oued	33.35	6.88	W		96	1	1	152	0	0	1	0	0	0	14	32.18	4,891	1.59	Sekour et al. (2014)
4	Albania	Drinos river	40.07	20.24	E		1,071	8	1	1,952	0	1	0	0	0	0	19	19.27	37,608	1.38	Paspali et al. (2013)
5	Greece	Antikythera	36.21	23.02	E	20	553	1	4	78	0	0	0	0	1	0	3	127.48	9,944	0.66	Alivizatos et al. (2005)
6	Spain	s´Espalmador	38.78	1.42	W	2	478	2	1	821	0	0	0	0	1	0	7	45.70	37,518	0.72	Guerra et al. (2014)
7	Spain	Formentera	38.68	1.56	W	83	478	2	1	173	0	0	0	1	0	0	5	79.92	13,826	0.97	Guerra et al. (2014)
8	Bulgaria	Kazanlak valley	42.67	25.42	E		755	30	1	18,421	0	1	0	0	0	0	16	22.87	421,245	1.45	Milchev (2015)
9	Bulgaria	Southeast region	42.30	27.21	E		639	32	1	24,107	0	1	0	1	0	0	24	18.53	446,609	1.30	Milchev et al. (2004)
10	Canary Islands	El Hierro	27.81	-17.97	W	269	352	1	5	1,053	0	1	0	0	0	0	4	29.57	31,134	0.69	Siverio et al. (2010)
11	France	Central Corsica	42.17	9.04	C	8,608	823	11	2	2,182	0	0	0	1	1	0	7	35.48	77,411	1.28	Libois (1984)
12	France	North Corsica	42.68	9.14	C	8,608	813	20	2	6,481	1	1	0	0	0	0	7	29.68	192,335	1.19	Libois (1984)
13	France	South Corsica	41.59	9.14	C	8,608	669	4	2	904	0	1	0	1	0	0	7	36.19	32,711	1.04	Libois (1984)
14	Cyprus	Paramytha	34.76	32.99	L	9,251	474	1	1	509	0	1	0	1	0	0	6	32.08	16,327	0.89	Own data
15	Cyprus	Profitis	35.02	34.04	L	9,251	526	1	1	416	0	1	0	1	0	0	4	37.29	15,513	0.91	Own data
16	Cyprus	Palae Pafos	34.77	32.41	L	9,251	483	1	1	115	1	1	0	0	0	0	4	57.34	6,594	0.46	Own data
17	Greece	Crete	35.34	25.08	E	8,261	628	1	1	276	0	1	0	1	0	0	5	25.70	7,093	0.75	Own data
18	Greece	Akrotiri	35.56	24.13	E	8,261	739	1	2	930	0	1	0	0	0	0	6	37.64	35,010	1.08	Obuch and Benda (2009)
19	Greece	Kolymvari	35.54	23.78	E	8,261	559	1	1	178	0	1	0	0	0	0	10	58.73	10,455	1.42	Own data
20	Greece	Archangelos	35.20	25.32	E	8,261	628	1	1	1,951	0	1	0	0	0	0	5	26.35	51,404	0.74	Own data
21	Egypt	Saqqara	29.86	31.22	E		125	1	1	230	0	1	1	0	0	0	3	48.46	11,146	0.62	Obuch and Benda (2009)
22	France	Perpignan	42.68	2.48	W		773	21	1	16,690	0	1	0	1	0	0	25	22.76	379,785	1.36	Libois (1984)
23	Greece	Evros delta	40.81	26.04	E		672	4	4	468	0	0	0	0	0	1	11	23.32	10,914	1.29	Alivizatos et al. (2005)
24	Greece	Chlemoutsi	37.89	21.14	E		777	1	1	481	0	1	0	1	0	0	9	25.05	12,048	1.46	Obuch & Benda (2009)
25	Greece	Messorachi	39.57	22.35	E		504	1	1	387	0	1	0	0	0	0	7	26.85	8,401	1.19	Bontzorlos et al. (2005)
26	Greece	Parnitha	38.25	23.69	E		549	1	4	237	0	1	0	0	0	0	13	35.66	6,364	1.43	Alivizatos et al. (2005)
27	Greece	Potidea	40.20	23.33	E		472	1	4	285	0	1	0	0	0	0	8	17.23	4,909	0.73	Alivizatos et al. (2005)
28	Greece	Parthenio	40.74	22.65	E		441	1	4	435	0	1	0	0	0	0	12	22.40	15,511	1.45	Alivizatos et al. (2005)
29	Greece	Samothraki	40.50	25.58	E	178	663	1	1	259	0	0	0	1	0	0	11	50.14	12,986	1.19	Own data
30	Greece	Stefanovikeio	39.46	22.74	E		539	1	1	574	0	1	0	0	0	0	12	67.00	38,455	1.62	Bontzorlos et al. (2005)
31	Greece	Vistonida Lake	41.03	25.08	E		642	1	1	1,811	0	0	0	0	0	1	14	21.71	40,561	1.31	Goutner & Alivizatos (2003)
32	Spain	Ibiza	39.03	1.57	W	572	478	1	2	2,516	0	1	0	1	0	0	9	35.26	88,704	1.37	Sommer et al. (2005)
33	Israel	Kibbutz	32.44	35.52	L		270	5	4	3,770	0	1	0	0	0	0	8	41.47	156,353	1.22	Tores et al. (2005)
34	Israel	Negev desert	30.97	34.40	L		90	1	1	989	0	0	1	0	1	0	13	34.07	33,696	1.44	Tores & Yom-Tov (2003)
35	Israel	Central region	31.42	34.81	L		463	18		7,967	0	1	0	0	0	0	11	46.65	371,674	1.29	Dor (1947)
36	Israel	Beit She'an	32.51	35.51	L		559	7	1	1,034	0	1	0	0	0	0	7	57.94	59,909	1.13	Charter et al. (2009)
37	Israel	Beit She'an	32.51	35.51	L		559	6	1	1,124	0	1	0	0	0	0	7	51.24	57,594	1.15	Charter et al. (2009)
38	Israel	Negev desert	30.29	35.07	L		31	1	1	243	0	0	1	0	0	0	9	39.61	9,625	1.01	Pokines & Peterhans (1997)
39	Israel	Northeast region	32.99	35.84	L		581	4	1	2,796	0	1	0	0	0	0	14	37.47	104,774	1.51	Obuch & Benda (2009)
40	Israel	Tel Aviv	32.11	34.85	L		579	1	2	706	1	1	0	0	0	0	6	38.48	27,168	1.20	Charter et al. (2007)
41	Israel	Beit She'an	32.51	35.51	L		559	7	1	1,017	1	0	0	0	0	0	7	50.30	51,152	1.41	Charter et al. (2009)
42	Italy	Siena	43.31	11.33	C		694	6	1	3,512	0	1	0	1	0	0	11	21.62	75,914	1.65	Lovari et al. (1976)
43	Italy	Siena	43.29	11.03	C		694	1	1	679	0	1	0	1	0	0	13	21.05	14,292	1.56	Pezzo & Morimando (1995)
44	Italy	Belagaio	43.08	11.22	C		695	1	1	567	0	1	0	1	0	0	16	20.58	11,666	1.87	Lovari et al. (1976)
45	Italy	Fuscaldo	39.41	16.03	C		900	1	1	731	0	1	0	1	0	0	10	29.16	21,318	1.61	Obuch & Benda (2009)
46	Italy	Po Plain	45.28	10.52	C		732	3	2	3,813	0	1	0	0	0	0	13	23.34	88,978	1.39	Bosè & Guidali (2001)
47	Italy	Pisa	43.78	10.29	C		824	13	1	4,029	0	1	0	1	0	0	16	22.65	91,252	1.29	Varuzza et al. (2001)
48	Jordan	Al-Sareeh	32.50	35.90	L		437	3	1	125	1	1	0	0	0	0	5	73.01	9,126	1.10	Rifai et al. (1998)
49	Jordan	Shaumari	31.55	37.17	L		43	2	1	282	1	0	1	0	0	0	5	61.34	17,297	0.51	Baker et al. (2005)
50	Greece	Karpathos	35.61	27.12	E	324	528	1	1	81	0	1	0	1	0	0	4	74.36	6,023	0.99	Own data
51	Greece	Korfu	39.79	19.92	E	324	787	1	3	1,233	0	1	0	0	0	0	12	51.99	64,100	1.07	Own data
52	Greece	Kos	36.90	27.29	E	324	534	4	1	781	0	1	0	0	0	0	8	39.27	30,672	1.35	Niethammer (1989)
53	Lebanon	Adloun Cave	33.41	35.27	L		684	1	3	1,934	1	1	0	0	0	0	16	29.10	56,276	1.61	Own data
54	Lebanon	Chaddra-Akkar	34.62	36.32	L		583	1	1	240	0	0	0	1	0	0	9	35.40	8,496	1.99	Abi-Said et al. (2014)
55	Lebanon	Sour	33.27	35.21	L		684	1	1	123	1	1	0	0	0	0	9	66.29	8,154	1.27	Obuch & Benda (2009)
56	Spain	North Menorca	40.04	4.02	W	668	607	2	1	550	0	0	0	1	0	0	9	42.89	23,590	1.39	De Pablo (2000)
57	Spain	South Menorca	39.85	4.24	W	668	602	7	1	3,005	1	1	0	0	0	0	9	39.77	119,507	1.04	De Pablo (2000)
58	Morocco	Chaouia	32.80	-8.00	W		360	3	1	4,890	0	1	0	0	0	0	15	21.34	104,349	1.00	Rihane (2003)
59	Morocco	Doukkala	32.76	-8.18	W		350	10	1	3,869	0	1	0	0	0	0	16	46.53	180,043	0.98	Rihane (2003)
60	Morocco	Lalla Mimouma	34.90	-6.07	W		520	6	1	803	0	1	0	1	0	0	4	32.63	26,205	0.81	Rihane et al. (2015)
61	Morocco	Mohammedia	33.67	-7.40	W		435	3	2	988	1	1	0	1	0	0	10	25.64	25,337	0.45	Rihane (2005)
62	Morocco	Oum	32.96	-7.81	W		360	2	1	1,063	0	1	0	0	0	0	14	27.13	28,839	1.07	Rihane (2003)

63	Syria	Al-Morizieb	32.72	36.03	L		241	3	1	131	0	1	0	0	0	0	6	30.45	3,990	1.18	Shehab (2005)
64	Italy	Grotta di Locoli	40.51	9.61	C	24,090	525	1	1	205	0	0	0	0	1	0	6	24.79	5,083	1.20	Di Russo (1987)
65	Syria	Azaz	36.59	37.11	L		468	1	1	621	0	1	0	1	0	0	9	33.70	20,925	1.03	Shehab & Al Charabi (2006)
66	Italy	Sicilia	37.92	13.34	C	25,711	571	14	1	2,781	0	1	0	0	0	0	6	21.06	58,557	1.13	Massa & Sarà (1982)
67	Italy	Sicilia	38.10	13.28	C	25,711	548	6	1	438	1	0	0	0	0	0	6	38.64	16,922	1.44	Massa & Sarà (1982)
68	Italy	Sicilia	37.90	13.40	C	25,711	571	10	1	1,343	0	1	0	1	0	0	5	17.88	24,018	1.09	Massa & Sarà (1982)
69	Syria	Kharabow	33.49	36.57	L		159	1	1	188	0	1	0	0	0	0	6	46.57	8,755	1.60	Shehab (2005)
70	Spain	Southern region	37.39	-4.46	W		504	26	1	12,487	0	1	0	0	0	0	10	21.81	272,387	1.19	Herrera & Jaksić (1980)
71	Spain	Fuentepiedra	37.13	-4.74	W		524	2	1	655	0	1	0	0	0	0	10	40.94	26,816	0.96	Vargas et al. (1982)
72	Spain	La Roca de la Sierra	39.10	-6.67	W		554	2	1	170	0	1	0	1	0	0	7	23.72	4,033	1.36	Amat & Soriguer (1981)
73	Spain	El Padul	37.03	-3.63	W		455	2	1	844	0	1	0	0	0	1	9	27.75	23,419	1.14	Vargas et al. (1982)
74	Syria	Qala'at Salah ad Din	35.60	36.06	L		698	4	1	2,331	0	1	0	1	0	0	14	40.55	94,526	1.33	Obuch & Benda (2009)
75	Serbia	Titel	45.20	20.30	E		609	1	1	231	0	1	0	0	0	0	10	25.08	5,793	1.45	Own data
76	Turkey	Aspendos	36.94	31.17	E		430	1	1	191	0	1	0	1	0	0	6	15.31	2,923	0.61	Own data
77	Turkey	Deveciuşaği	36.76	35.63	L		784	1	1	193	0	1	0	0	0	0	6	28.89	5,577	1.30	Obuch & Benda (2009)
78	Turkey	Girmeler	36.59	29.38	E		756	1	1	193	0	1	0	1	0	0	6	20.67	3,989	0.79	Own data
79	Turkey	Harran	36.86	39.03	L		349	2	1	1,141	0	1	0	0	0	0	11	35.60	40,621	1.05	Horáček et al. (1995)
80	Turkey	Narlikuyu	36.44	34.11	L		557	1	1	922	0	0	0	1	0	0	19	25.14	23,177	1.94	Own data
81	Turkey	Menderes Delta	37.55	27.23	E		704	1	1	296	0	1	0	0	0	0	7	19.30	5,714	1.06	Brinkmann et al. (1991)
82	Turkey	Samandağ	34.47	9.51	L		279			133	0	1	0	0	0	0	6	53.63	7,133	0.86	Hoppe (1986)
83	Turkey	Lake Bafa	36.07	35.98	E		853	1	1	151	0	1	0	1	0	0	5	21.19	3,200	0.95	Kasparek (1988)
84	Turkey	Milet	37.51	27.52	E		704	2	1	576	0	1	0	0	0	0	8	17.75	10,222	0.97	Niethammer (1989)
85	Tunisia	Bou	37.53	27.28	C		704	2	1	1,274	0	0	0	0	1	0	6	16.87	21,493	1.44	Leonardi & Dell'Arte (2006)
